# Tissue‐Engineered Cochlear Fibrosis Model Links Complex Impedance to Fibrosis Formation for Cochlear Implant Patients

**DOI:** 10.1002/adhm.202300732

**Published:** 2023-06-22

**Authors:** Simone R. de Rijk, Alexander J. Boys, Iwan V. Roberts, Chen Jiang, Charlotte Garcia, Róisín M. Owens, Manohar Bance

**Affiliations:** ^1^ Cambridge Hearing Group Cambridge CB2 8AF UK; ^2^ Department of Clinical Neurosciences University of Cambridge Cambridge CB2 3 EB UK; ^3^ Department of Chemical Engineering and Biotechnology University of Cambridge Cambridge CB3 0AS UK; ^4^ Department of Electronic Engineering Tsinghua University Beijing 100190 P. R. China; ^5^ Medical Research Council Cognition and Brain Sciences Unit University of Cambridge Cambridge CB2 7EF UK

**Keywords:** bioelectronics, biological circuit modelling, electrochemical impedance spectroscopy, electrodes, tissue engineering

## Abstract

Cochlear implants are a life‐changing technology for those with severe sensorineural hearing loss, partially restoring hearing through direct electrical stimulation of the auditory nerve. However, they are known to elicit an immune response resulting in fibrotic tissue formation in the cochlea that is linked to residual hearing loss and suboptimal outcomes. Intracochlear fibrosis is difficult to track without postmortem histology, and no specific electrical marker for fibrosis exists. In this study, a tissue‐engineered model of cochlear fibrosis is developed following implant placement to examine the electrical characteristics associated with fibrotic tissue formation around electrodes. The model is characterized using electrochemical impedance spectroscopy and an increase in the resistance and a decrease in capacitance of the tissue using a representative circuit are found. This result informs a new marker of fibrosis progression over time that is extractable from voltage waveform responses, which can be directly measured in cochlear implant patients. This marker is tested in a small sample size of recently implanted cochlear implant patients, showing a significant increase over two postoperative timepoints. Using this system, complex impedance is demonstrated as a marker of fibrosis progression that is directly measurable from cochlear implants to enable real‐time tracking of fibrosis formation in patients, creating opportunities for earlier treatment intervention to improve cochlear implant efficacy.

## Graphical abstract

This study focuses on the development of a tissue‐engineered system for mimicking fibrotic response occurring after cochlear implant placement, enabling real‐time monitoring of implant electrical response. A representative circuit model, informed by electrical impedance spectroscopy, is constructed for this tissue‐engineered system, and a new electrical fibrosis marker is found that corroborates with observations during cochlear implant healing in patients.

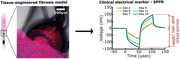

## 1 Introduction

Hearing loss affects 20% of the world's population with an ≈5% needing clinical intervention.^[^ ^1^ ^]^ Cochlear implants (CIs) are life‐changing technology that allows people with severe hearing loss to hear and achieve speech perception.^[^ ^2^ ^]^ CIs, arguably the most successful neural prostheses to date, transform sounds into electrical pulses that directly stimulate the auditory nerve.^[^ ^3^ ^]^ The intracochlear^[^ ^4^, ^5^ ^]^ multi‐electrode array of CIs takes advantage of tonotopic, frequency‐dependent, organization of the cochlea by electrically stimulating different parts of the auditory nerve processes to convey different sounds.^[^ ^3^ ^]^ However, these implants are known to cause fibrosis when placed, which can limit their efficacy long‐term.

Implants are known to elicit an inflammatory response, associated with fibrotic encapsulation.^[^ ^6^, ^7^, ^8^ ^]^ Fibrosis is of particular concern for neural implants, as the fibrotic capsule can limit electrical signal transduction to surrounding tissues.^[^ ^8^, ^9^, ^10^, ^11^ ^]^ For CIs, the inflammatory response is driven by mechanical trauma during insertion, which results in protein absorption, particularly fibrin,^[^ ^9^, ^12^, ^13^, ^14^, ^15^ ^]^ extracellular matrix (ECM) deposition, and subsequent cell‐mediated contraction into a dense fibrotic capsule around an implant.^[^ ^11^, ^16^, ^17^ ^]^ This process is initiated by various immune cells, such as macrophages, before infiltration by fibroblastic cells that lay down further ECM.^[^ ^9^, ^12^, ^13^, ^14^, ^15^ ^]^ The extent of new tissue formation in the cochlea can vary from a thin fibrous sheath surrounding the electrode array, to new bone formation.^[^ ^18^, ^19^, ^20^, ^21^, ^22^ ^]^ This overall inflammatory response to CI insertion has been associated with the loss of intracochlear hair cells and auditory neurons^[^ ^4^, ^9^, ^23^, ^24^, ^25^ ^]^ and restriction of basilar membrane vibration,^[^ ^26^ ^]^ which subsequently results in residual acoustic hearing loss.^[^ ^18^, ^23^, ^27^, ^28^, ^29^ ^]^ Studying cochlear fibrosis can inform new treatments, such as efficacy for drug‐eluting electrode arrays, and may provide insight into current treatments to prevent fibrosis^[^ ^4^, ^5^, ^27^, ^30^, ^31^, ^32^ ^]^ or emerging treatments, such as cell and gene therapies and optogenetic stimulation.^[^ ^33^, ^34^ ^]^

Despite the relevance of intracochlear fibrosis to residual hearing loss, we possess few methods for tracking fibrosis in CI patients.^[^ ^18^ ^]^ One indirect method is the measurement of contact “impedances,” an increase of which has been associated with fibrotic tissue formation and residual hearing loss in patients.^[^ ^5^, ^19^, ^35^, ^36^, ^37^ ^]^ While not actual electrical impedance measurements, contact impedances are voltage responses at a single timepoint to a biphasic charge‐balanced current pulse, normalized to the amplitude of input current.^[^ ^38^ ^]^ These have been investigated in preclinical models with posthumous evaluation^[^ ^4^, ^5^ ^]^ but lack real‐time measurements and vary significantly from human anatomy. Other studies have explored fibrosis using 2D in vitro models,^[^ ^36^, ^39^ ^]^ but these studies lack the complexity of the 3D matrix deposition and contraction seen in vivo.^[^ ^40^ ^]^ Tissue‐engineered models provide a potential in‐roads for examining the relationship of fibrosis to electrical measurements, given their capabilities for simulating cellular phenomena in 3D, such as tissue contraction.^[^ ^41^, ^42^ ^]^ Further, these models could be coupled to clinical‐grade implants, where the overall frequency and responses of the model can be studied. While replication of the immune system is challenging in vitro, resultant tissue‐engineered ECMs can possess similar properties to in vivo fibrotic tissue by harnessing the capabilities of cells to remodel tissue‐engineered matrices.^[^ ^43^, ^44^ ^]^ Electrochemical impedance spectroscopy (EIS) has long been used to measure cell and tissue behavior such as proliferation, differentiation, cell adhesion, detect various forms of malignancies, monitor 3D cell cultures, and detect liver fibrosis.^[^ ^45^, ^46^, ^47^ ^]^ By measuring frequency response of impedance, EIS provides higher‐content information on tissues, lending promise for tracking fibrosis progression.

In this study, we investigate complex impedance as a biomarker for fibrosis progression by developing a 3D tissue‐engineered model of cochlear fibrosis. We replicate the intracochlear fibrotic environment by encapsulating clinical‐grade CI electrode arrays inside tissue‐engineered fibroblast‐seeded fibrin gel constructs. We show significant and consistent changes in complex impedance over time, with which we produce a realistic electrical circuit model for fibrosis development. We also utilize full voltage waveform measurements to propose an electrical marker of fibrosis development that could be implemented clinically, finding similar electrical behavior in our measurements of patient samples. The results presented in this study and the markers we propose will enable us to track cochlear fibrosis progression in real‐time, allowing for earlier treatment intervention for combating residual hearing loss for CI patients.

## 2 Results

### 2.1 Development of a Tissue Engineered Model of Cochlear Implant Fibrosis

**Figure 1:**
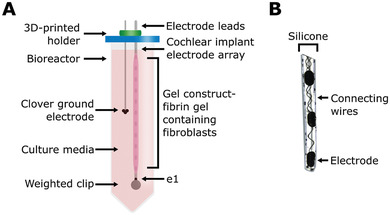
Schematic of 3D bioreactor setup. A) Schematic of the tissue‐engineered cochlear fibrosis model construct including a cochlear implant electrode array encapsulated with a fibrin gel with 3D‐seeded fibroblasts. e1 represents the first/most apical electrode. B) Image of the three apical electrode contacts including connecting wires.

We modeled cochlear fibrosis by producing a fibrous sheath around a clinical‐grade cochlear electrode array. To generate this model, we injected molded fibrin gels containing fibroblasts into a 3D‐printed mold, with the cochlear electrode array centered on the axis. These electrode arrays with cell‐seeded gel constructs were suspended in culture media inside a conical bioreactor, to set the electrode array and ground electrode location for consistent electrical measurement (**Figure** **1**). Fibrin was chosen as the biological scaffold as it is the provisional matrix laid down during wound healing,^[^ ^48^ ^]^ both post‐implantation of cochlear implants^[^ ^9^, ^12^, ^13^, ^14^, ^15^ ^]^ and other implanted biomaterials scenarios.^[^ ^49^ ^]^ Given the composition of the constructs, cells were expected to interact with and contract fibrin gels into a denser conformation around the array.^[^ ^42^ ^]^ To promote increased interaction between fibroblasts and gel, a contractile medium was formulated, along with media supplementation of TGF‐*β*1 to promote fibroblast differentiation into a more fibrotic‐like, contractile phenotype.^[^ ^49^, ^50^, ^51^ ^]^ Images were captured to track contraction throughout the experiment. We measured EIS and voltage waveforms at six timepoints over the course of 14 days (days 2, 4, 7, 9, 11, and 14) and took concurrent images beginning on day 0 (Figure 1A; Figure S1A,B, Supporting Information). To examine the effects of electrical stimulation, from our measurement criteria, we also utilized an unstimulated control.

**Figure 2:**
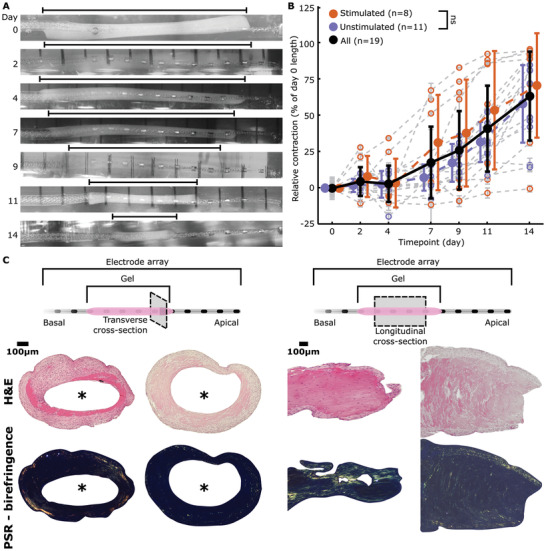
Contraction and histology analysis of the constructs. A) Representative image set showing contraction of the construct over the course of the experiment. The length of the construct was calculated using the known mid‐to‐mid contact spacing of the electrode arrays to calculate the scale of the images for each electrode array and timepoint separately, which was then used to calculate the length of the construct. B) Relative contraction of the constructs, normalized to day 0 absolute length, over time. Single datapoints are shown in grey lines with open circles. Mean ± standard deviation is shown for the stimulated and unstimulated groups (ns = not significant, univariate n‐way ANCOVA) in bold as well as all constructs. Relative contraction is significant over time (*p* < 0.001, univariate n‐way ANCOVA) with the inflection point between days 7 and 9 (Tukey's post hoc test). C) Hematoxylin & eosin (H&E) and polarized picrosirius red (PSR) stained histology slices, transverse and longitudinal sections, of two constructs. “*” represents the location of the electrode array. H&E staining shows higher density of cells at the lateral and medial edges of the construct. PSR reveals birefringence and thus collagen formation. No differences between the stimulated and unstimulated constructs are visible.

We observed considerable axial contraction of the constructs (**Figure** **2**). To correct for variance in absolute length, we analyzed the contraction as a relative measure normalized to day 0 construct length (Figure 2B). Significant relative contraction as a function of time was found (F = 22.25, *p* < 0.001, *df* = 6, *n* = 133, univariate *n*‐way analysis of variance (ANOVA), experiment number as random factor), with a mean contraction of 60% ± 35% (standard deviation). The inflection point, as determined by a Tukey's post hoc test, was found between days 7 and 9.

Next, we performed histology to retrieve information on cellular orientation and extracellular matrix morphology (Figure 2C; Figure S2, Supporting Information). Hematoxylin and Eosin (H&E) staining revealed a higher lateral density of cells with denser medial ECM, indicating cellular repositioning with respect to available nutrients. We performed picrosirius red (PSR) staining for oriented fibrillar collagen^[^ ^52^, ^53^ ^]^ to examine for collagen orientation. Some coloration is evident (Figure 2C), indicating that the fibroblasts are producing dense, fiber‐like collagen bundles, most likely via mechanical boundary conditions,^[^ ^54^ ^]^ which are inherently applied by the presence of the CI array. We also utilized a Ki‐67 stain, a marker of cell proliferation,^[^ ^55^ ^]^ to confirm that cells within the constructs where proliferating in all cases (Figure S2, Supporting Information).

Use of electrical stimulation as a method to prevent CI fibrosis has sparked recent interest.^[^ ^35^, ^36^, ^56^, ^57^ ^]^ Therefore, we tested the effect of stimulation on contraction, while correcting for additional sources of variation. No significant effect of stimulation was found (F = 1.59, *p* = 0.23, *df* = 1, univariate n‐way ANOVA, relative contraction on day 14‐dependent variable, stimulation, electrode design‐fixed factors, and experiment number‐random factor). We also did not observe any differences from our histological analysis (H&E, PSR, Ki‐67). To confirm these similarities, we performed a Hoechst fluorescence assay to quantify DNA at day 14 (Figure S3, Supporting Information). No significant effect of stimulation was found (F = 0.08, *p* = 0.78, *df* = 1, univariate n‐way ANOVA, DNA per µg dry weight as dependent variable (*n* = 10), stimulation‐fixed factor (*n* = 4 simulated, *n* = 6 unstimulated), experiment number‐random factor (*n* = 2 Exp1, *n* = 4 Exp2, *n* = 4 Exp3)). These results agree with studies investigating the effects of early switch‐on and more extensive stimulation post‐operatively, which show little effect on long‐term markers of fibrosis formation.^[^ ^58^, ^59^ ^]^

**Figure 3:**
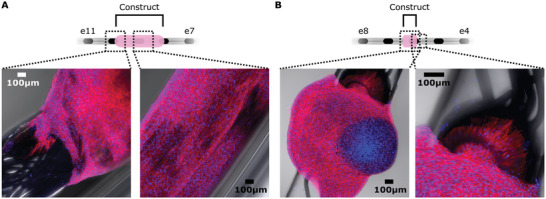
Confocal fluorescence imaging of the construct. 3D orientation of the construct, as fixed on day 14, related to the electrode array. The nuclei are stained blue via DNA staining with Hoechst 33258, while actin was stained with phalloidin‐iFluor 594 showing in red. A) Edge and mid‐construct images without stimulation. B) Total and close‐up of a stimulated construct. Both constructs show attachment of the cells on the electrode surfaces. Actin fibers in the cells can be seen spreading out over the surface of the electrodes and numerous cells are attached to a singular electrode alone.

Given that constructs axially contract, we found in some cases, constructs would contract away from electrodes that were covered on day 0. To understand the 3D structure of the constructs relative to positioning along the arrays, samples (*n* = 2, 1 stimulated, 1 unstimulated) were stained for DNA and actin. The edge and center of the constructs are visible, showing dense cells attached to the arrays (**Figure** **3**A). This allowed us to visualize areas that had become uncovered during contraction, where we observed no evidence of construct remnants. We also observed cellular spreading on an exposed electrode at the trailing edge of the construct (Figure 3B). This observation indicates that cells can adsorb directly onto electrodes, potentially effecting electrode–electrolyte (EE) interface during stimulation. However, as no residual construct remained in areas of arrays that had become uncovered during contraction, this interface is potentially recoverable.

Within the statistical tests described in this section, an effect of experiment number was found on relative contraction and DNA quantification (Figures S1C and S3, Supporting Information), possibly indicating some variance within the fibroblast cell line used for this study.

### 2.2 Electrochemical Impedance Spectroscopy Shows Significant Changes in the Bulk of the Gel

**Figure 4:**
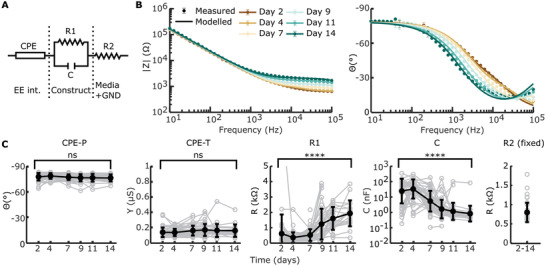
Complex impedance measured with electrochemical impedance spectroscopy (EIS). EIS was measured on all electrodes, regardless of having an open circuit (e.g., air bubble or broken electrode). The exclusion criteria, as described in the materials & methods section, led to *n* = 231 recordings with construct and *n* = 153 without construct being included (of a combined total of *n* = 528 recordings). A) Proposed equivalent circuit of the 3D bioreactor model with a constant phase element (CPE) representing the electrode‐electrolyte (EE) interface, a resistor (*R*_1_) in parallel with a capacitor (C) representing the bulk of the construct, and an additional resistor (*R*_2_) representing the media and ground (GND). B) Absolute impedance magnitude and phase angle of an example electrode over time, showing both measured and modeled values. Measured data are shown as mean ± standard deviation. C) Modeled circuit elements over time of all timepoints and electrodes with construct (and thus modeling fibrosis) on the electrode. Individual data are shown in grey. The arithmetic mean ± standard deviation is shown in bold black, except for C where the geometric mean and standard deviation is shown. CPE‐P and CPE‐T show no significant (ns) changes from day 2 to day 14 (univariate n‐way ANOVA, Tukey's post hoc test). *R*_1_ shows a significant increase from day 2 to day 14 (^****^ *p* < 0.001, univariate n‐way ANOVA, Tukey's post hoc test), while C shows a significant decrease (^****^ *p* < 0.001, univariate n‐way ANOVA, Tukey's post hoc test).

We hypothesized that complex impedance would change as measured via EIS with cellular contraction and construct remodeling. To test this hypothesis, we tracked complex impedance spectra over six timepoints to day 14. These spectra were fitted to a circuit model (**Figure** **4**A), consisting of a constant phase element (CPE) representing EE interface, a resistor (R_1_) in parallel with a capacitor (C) representing the bulk of the construct, and an additional resistor (R_2_) representing the resistance of the media and ground. Since we did not expect a major contribution to overall impedance with changes in cell media and pathway to ground, R_2_ was fixed based on the earliest available timepoint for each electrode. The EE interface and bulk of the construct have been hypothesized to change during cochlear fibrosis.^[^ ^35^, ^36^, ^39^, ^60^, ^61^ ^]^ So, these elements were fitted without constraints. The average weighted sum‐of‐squares, proportional to the average percentage error between original and fitted data, was <1% for most fittings and at least <5% for all fittings (Figure S4A, Supporting Information). An example of impedance magnitude and phase angle over time, for both measured and modeled data, for 1 electrode with the construct on throughout the experiment can be seen in Figure 4B and without construct in Figure S4B (Supporting Information). An increase in absolute impedance magnitude is seen at higher frequencies (>10 kHz), while phase angle decreased across most frequencies in this example.

Fitted circuit elements over time with construct on showed CPE phase (CPE‐P) and magnitude (CPE‐T) stay constant, while circuit element R_1_ increased and C decreased (Figure 4C). This change is not seen for electrodes without constructs on them (Figure S4C). R_1_ showed a large significant effect of time (F = 21.51, *p* < 0.001, *df* = 5), with the inflection point between days 7 and 9 as revealed by Tukey's post hoc test, and overall significant change between day 2 and day 14 (*p*<0.001). C also showed a large significant effect of time (F = 15.12, *p* < 0.001, *df* = 5), with the inflection point between days 4 and 7 as revealed by Tukey's post hoc test, and an overall significant decrease between days 2 and 14 (*p* < 0.001). CPE‐P and CPE‐T remained largely. When comparing to fitted circuit elements for electrodes without construct, no significant effect of time is found for CPE‐P (F = 1.06, *p* = 0.38, *df* = 5), CPE‐T (F = 0.79, *p* = 0.56, *df* = 5), R_1_ (F = 0.78, *p* = 0.56, *df* = 5), and C (F = 1.85, *p* = 0.11, *df* = 5). Overall, these data suggest changes in EIS can be explained by an increase in resistance and decrease in capacitance of the bulk of the construct with no significant changes in EE interface seen.

A commonly studied circuit to model contact impedances in relation to cochlear fibrosis was introduced by Tykocinski et al. and includes a resistor in parallel with a capacitor representing EE interface and a single resistor in series representing the bulk of tissue (Figure S5A, Supporting Information).^[^ ^62^ ^]^ This circuit is extracted from a voltage waveform (contact impedance timepoints) and models access resistance, initial increase in voltage at the start of the waveform, and polarization impedance, the capacitive build‐up after access resistance.^[^ ^62^ ^]^ Changes in polarization impedance have since been linked to protein adsorption (increase) and resorption (decrease) on the electrode.^[^ ^35^, ^39^, ^57^ ^]^ Changes in access resistance are more commonly associated with changes in bulk tissue surrounding the electrode, where an increase in access resistance is linked to an increase in tissue formation.^[^ ^36^, ^39^, ^56^, ^60^ ^]^ However, changes are not specific to new tissue formation only, as an increase in access resistance has also been associated with electrode‐modiolus distance, translocation of the electrode from one scala to another intracochlearly, extracochlear electrodes, and electrode failure.^[^ ^63^, ^64^, ^65^, ^66^, ^67^ ^]^ We fitted this circuit to our example data (Figure 4B; Figure S5B, Supporting Information) mainly showing a large error in phase angle for complex impedance. Average weighted sum‐of‐squares was >10% in all six timepoints (Figure S5C, Supporting Information), suggesting this circuit is too simple to model complex impedance for our model of fibrosis.

### 2.3 Contact Impedances and Second Phase Peak Ration (SPPR) of Voltage Waveforms Increase Significantly Over Time

**Figure 5:**
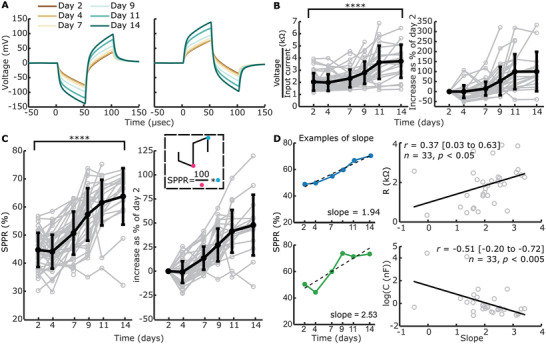
Measured voltage waveforms, contact “impedances” and SPPR over time. Voltage waveforms were only measured when EIS measurements were included and a single pulse did not elicit high voltage waveform responses, leading to *n* = 221 with construct and *n* = 115 without construct. A) Example mean voltage waveforms over time for the same electrode as in Figure 4B, with a cathodic‐leading biphasic pulse and anodic‐leading biphasic pulse as an input. B) Absolute and relative contact “impedances” over time. Individual traces are shown in grey, while mean ± standard deviation is shown in bold black. Absolute contact “impedances” significantly increased over time from day 2 to day 14 (^****^ *p* < 0.001, univariate n‐way ANOVA, Tukey's post hoc test). C) Absolute and relative SPPR shown over time. Individual traces are shown in grey, while the mean ± standard deviation is shown in bold black. A schematic of how the SPPR is calculated is shown (second phase peak as a percentage of the first phase peak). Absolute SPPR significantly increased over time from day 2 to day 14 (^****^ *p* < 0.001, univariate n‐way ANOVA, Tukey's post hoc test). (D) Example of linear function fitting to SPPR over time including the output slope are shown for a relatively good (blue) and bad (green) fit. The linear function is shown as a dashed black line. The slope of the linear fit is significantly positively correlated with *R*_1_ as fitted with EIS and significantly negatively correlated with C as fitted with EIS (Pearson's correlation coefficient).

To translate the changes in complex impedance to an electrical measurable in patients, we measured voltage waveforms at all timepoints for electrodes with and without construct on (**Figure** **5**A; Figure S6A, Supporting Information). Generally, an increase in voltage over time is observed with construct on the electrode, while no changes are seen without construct on the electrode. When the construct contracts off an electrode, the voltage waveform was seen to normalize back to the level of the waveform at day 2 (Figure S6B).

Contact impedances, voltage at the end of the first phase of a cathodic‐leading pulse normalized to the input current, were calculated with (Figure 5B) and without (Figure S6C, Supporting Information) constructs on the electrode. Contact impedances with construct on the electrode were normalized to day 2. Contact impedances significantly increased over time when construct was on the electrode (F = 23.91, *p* < 0.001, *df* = 5, univariate n‐way ANOVA corrected for experiment number (random factor)), with inflection point between days 7 and 9 as revealed by Tukey's post hoc test, and an overall significant change between days 2 and 14 (*p* < 0.001). Without construct on the electrode, no significant changes were seen over time (F = 1.32, *p* = 0.26, *df* = 5).

We hypothesized that with an increase in R_1_ and a decrease in C over time for electrodes with construct on, ratio of the second peak as a percentage of the first peak would change over time, as the contribution of capacitive discharge to the second phase peak would decrease. Therefore, we calculated the second phase peak ratio (SPPR) as shown in Figure 5C, which describes second peak voltage as a percentage of the first peak voltage. The SPPR increased significantly over time when the construct was on the electrode (F = 38.05, *p* < 0.001, *df* = 5, univariate n‐way ANOVA corrected for experiment number (random factor)), with the inflection point between days 4 and 7 (Tukey's post hoc test). Here, a significant difference was found between days 2 and 14 (*p* < 0.001). Without construct on the electrode (Figure S6D, Supporting Information), no significant changes were seen with time (F = 1.07, *p* = 0.38, *df* = 5).

To compare change in SPPR over time with a single measure to EIS‐fitted circuit elements R_1_ and C, we calculated slope of SPPR over time with a linear function. Two examples of such slopes can be seen in Figure 5D, with both SPPR that shows a linear increase over time and one that does not. We only fitted data to a linear function when >3 datapoints and datapoints after day 7 (inflection point of R_1_) were available, leading to *n* = 33 slopes. These slopes were correlated with the final available timepoint used for the linear fit (Figure 5D) of EIS‐fitted circuit elements. A significant positive correlation was found between the SPPR and R_1_ (Pearson's *r* = 0.37 (95% CI: 0.03 to 0.63), *p* < 0.05, *n* = 33), while a significant negative correlation was found between SPPR and C (Pearson's *r* = −0.51 (95% CI: −0.20 to −0.72), *p* < 0.005, *n* = 33).

### 2.4 Voltage Waveform‐Fitted Circuit Elements Correlate Significantly with EIS‐Fitted Circuit Elements

**Figure 6:**
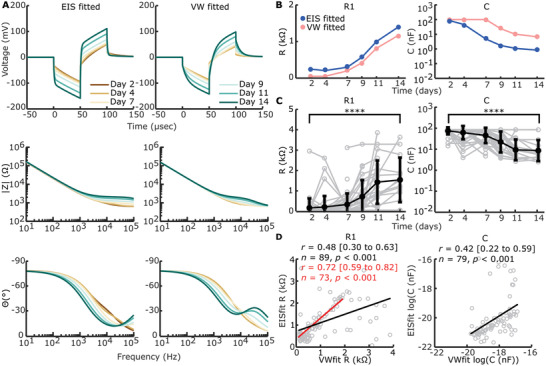
Reverse fitting of voltage waveforms (VW) to electrical circuit. A) EIS‐fitted and VW‐fitted voltage waveforms (top row), absolute impedance magnitude (middle row), and phase angle (bottom row) of electrical circuit in Figure 4A on example data shown in Figures 4B and 5A. B) Example of direct comparison between EIS‐fitted (blue) and VW‐fitted (pink) circuit element sizes for the example shown in (A). C) VW‐fitted circuit elements over time of all timepoints and electrodes with construct on the electrode (*n* = 193). Individual data is shown in grey. The arithmetic mean ± standard deviation is shown in bold black, except for C where the geometric mean and standard deviation are shown. *R*_1_ shows a significant increase from day 2 to day 14 (^****^ *p* < 0.001, univariate n‐way ANOVA, Tukey's post hoc test), while C shows a significant decrease (^****^ *p* < 0.001, univariate n‐way ANOVA, Tukey's post hoc test). D) Correlation between VW‐fitted and EIS‐fitted *R*_1_ and *C*, excluding uncapped values (including *n* = 89 for *R*_1_ and *n* = 79 for *C*, compared to *n* = 193 for both) as part of the bimodal distribution seen in Figure S7C (Supporting Information). A significant but modest correlation was found for both circuit elements (Pearson's correlation coefficient). For *R*_1_, the correlation is stronger when VW‐fitted elements >2 kΩ are excluded, suggesting outliers are more likely with *R*_1_ > 2 kΩ in VW‐fitting.

To expand information extraction from voltage waveforms, we reverse fitted (voltage waveform (VW) fitted) (Figure S7A, Supporting Information) our chosen circuit (Figure 4A) to the voltage waveforms (**Figure** **6**A). R_1_ and C show similar trends, yet capacitance is higher for the VW‐fitted example. Additionally, C is capped at its upper limit (10^2^ nF) for days 2 through 7.

CPE‐P and CPE‐T were fixed based on day 2 values in addition to fixed R_2_ values, and so only data with EIS fitting available on day 2 was included (Figure 6C). R_1_ increased significantly over time (F = 17.83, *p* < 0.001, *df* = 5, univariate n‐way ANOVA corrected for experiment number (random factor)), whilst C decreased significantly over time (F = 23.98, *p* < 0.001, *df* = 5). The inflection point, as shown by Tukey's post hoc test, was in between days 7 and 9 for R_1_ and days 4 and 7 for C. Significant differences from days 2 to 14 were present for both R_1_ (*p* < 0.001) and C (*p* < 0.001).

A percentage of VW‐fit output shows capped values where R_1_ caps its lowest bound of 50 Ω and C caps its upper bound of 10^−7^ F. Capping mainly happens when the voltage waveform peak is at its lowest, since 60.6% of the output values is capped in at least one element over all timepoints, whilst from days 9 to 14, only 21% of the VW‐fittings is capped (Figure S7B, Supporting Information). This leads to a bimodal distribution for output values of VW‐fitted R_1_ and C with the element bounds used (Figure S7C, Supporting Information). Widening the element bounds, however, leads to capping at both bounds for both circuit elements (Figure S7D, Supporting Information). To compare EIS‐fitting with VW‐fitting we correlated EIS‐fitted values of circuit elements R_1_ and C to VW‐fitted values of the same electrode and timepoint. A significant positive correlation was found between EIS‐fitted R_1_ and VW‐fitted R_1_ (Pearson's *r* = 0.48 (95% CI: 0.30 to 0.63), *p* < 0.001, *n* = 89). However, outliers were seen when VW‐fitted R_1_ reached >2 kΩ. Excluding VW‐fitted R_1_ > 2kΩ showed a stronger correlation between VW‐fitted and EIS‐fitted R_1_ (Pearson's *r* = 0.72 (95% CI: 0.59 to 0.82), *p* < 0.001, *n* = 73). A significant positive correlation was also found between EIS‐fitted C and VW‐fitted C (Pearson's *r* = 0.42 (95% CI: 0.22 to 0.59), *p* < 0.001, *n* = 79).

### 2.5 Changes in Contact Impedances and SPPR in CI Patients Postoperatively are in Line with Changes Found in the Tissue Engineered Model

**Figure 7:**
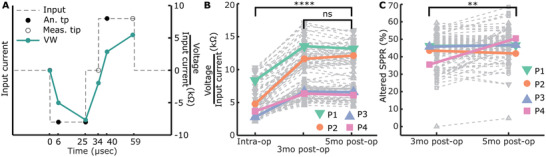
Contact “impedances” and altered SPPR of four recently implanted CI patients. A) Schematic of input current (dashed line), measured timepoints (circles), and analyzed timepoints (filled circles). An example response from a patient is shown in turquoise. B) Contact “impedances”, as measured at the end of the first phase (25 µs), intraoperatively, 3 months postoperatively, and 5 months postoperatively for four patients. Individual data are shown in grey, the mean of each patient is shown in bold. A significant increase in contact “impedances” is seen from intra‐op to post‐op on group level (^****^ *p* < 0.001, univariate n‐way ANOVA, Tukey's post hoc test), but not from 3 to 5 months postoperatively (ns = not significant, univariate n‐way ANOVA, Tukey's post hoc test). C) Altered SPPR (6 µs into each phase) is shown for two post‐operative timepoints. Individual data are shown in grey, the mean of each patient is shown in bold. A significant increase in SPPR is seen from 3 to 5 months post‐op on group level (^**^ *p* < 0.01, univariate n‐way ANOVA, Tukey's post hoc test).

Based on our findings of SPPR changes in our tissue‐engineered model, we wanted to test this marker in recently implanted CI patients. We used the CI company's software function to measure mutliple timepoints along voltage waveform response, to measure an altered version of SPPR (6 µs into each phase) as well as compare this to the contact impedances (**Figure** **7**A) over 2 and 3 timepoints, respectively, in four patients. We assumed no or little fibrosis was present before cochlear implantation, since these were new CI patients, and at least some fibrosis formation to occur within 5 months postoperatively. It should be noted that, given the inability of currently‐used diagnostics to monitor fibrosis progression, we have no independent information about fibrosis status at the collected timepoints.

Contact impedances showed a significant increase from intraoperative to postoperative timepoints (F = 139.1, *p* < 0.001, *df* = 2, *n* = 264 datapoints across four patients, univariate n‐way ANOVA, *p* < 0.001 Tukey's post hoc test), when correcting for patient as a random factor and electrode number as a fixed factor (Figure 7B). No significant effect of patient (F = 2.36, *p* = 0.07, *df* = 3–4 patients) nor electrode number (F = 0.35, *p* = 0.997, *df* = 21,22 electrodes) were found. Postoperative contact impedances at 3 and 5 months were not significantly different from each other (*p* = 0.995, Tukey's post hoc test).

SPPR was only available for two postoperative timepoints (Figure 7C). The altered SPPR showed a significant increase over time (F = 6.83, *p* < 0.01, *df* = 1, *n* = 176 datapoints across four patients, univariate *n*‐way ANOVA), when correcting for patient and electrode number. No significant effects for patient (F = 1.99, *p* = 0.118, *df* = 3–4 patients) or electrode number (F = 1.41, *p* = 0.120, *df* = 21–22 electrodes) were found. The largest increase in mean altered SPPR is seen for patient 4 (P4, Figure 7C), while a modest increase is seen for P1 and P3 and a modest decrease for P2. While an increase in altered SPPR is found for all 22 electrodes for P4, a variation of increase and decrease is found for the other three patients when looking at electrode level changes.

Mean comfortable loudness levels (MCLs), expressed in the log‐scale unit of current levels (CLs), were available for the 3 and 5 months postoperative timepoints for a subset of electrodes. MCLs are complex and likely to vary due to patient‐related factors such as, but not limited to, auditory nerve survival and central factors. Therefore, we looked at the relationship between change in SPPR and contact impedances with change in MCLs, as to correct for between‐patient differences in absolute MCL. When looking at changes in contact impedances and SPPR (Figure S8, Supporting Information) from 3 to 5 months per electrode, no significant correlations were found with changes in MCLs (change in contact impedances: Pearson's *r* = 0.23 (95% CI: −0.15 to 0.54), *p* = 0.23, *n* = 30, change in altered SPPR: Pearson's *r* = −0.32 (95% CI: −0.61 to 0.05), *p* = 0.09, *n* = 30). The negative correlation between change in altered SPPR with change in MCL per electrode was mainly driven by three datapoints of P4 that showed a large positive change in altered SPPR and a decrease in MCL. An overview of all the patient data can be found in Table S1 (Supporting Information).

## 3 Discussion

In this study, we tissue engineered a 3D model of cochlear fibrosis that behaves similarly to data we collected from a postoperative population of patients with cochlear implants. This model was designed to improve our understanding of the fibrotic response that occurs during cochlear implantation and ideally will be used in conjunction with large‐scale human data collection and animal models to improve outcomes for patients experiencing the effects of fibrosis from the placement of a cochlear implant. We used a tissue‐engineered, cell‐seeded gel to simulate the electrical environment of a fibrosing cochlear implant on a clinical cochlear electrode array. We analyzed these data both biologically and electrically to confirm the usefulness of this system as a model for cochlear fibrosis. Finding that we could recreate some of the conditions that we observed in a patient population, we developed a new marker based on our electrical data that was also found to increase in our postoperative patient‐derived data at group level. Cochlear implants are known to cause fibrosis formation in the cochlea that can lead to residual hearing loss for cochlear implant patients.^[^ ^4^, ^9^, ^23^, ^24^, ^25^, ^26^ ^]^ An electrical marker of fibrosis progression could create an early window for treatment intervention to reduce the residual hearing loss for patients.

Our tissue‐engineered model of cochlear fibrosis has the advantage of including the electrode array that is used in the clinical setting for CI patients, as well as incorporating the 3D aspect of fibrous tissue encapsulation that is known to behave differently from 2D tissue.^[^ ^40^ ^]^ Using this model, we were able to examine some cellular behaviors for which the field has only been able to previously speculate.^[^ ^39^, ^68^ ^]^ We found attachment of the fibroblasts to the electrode surfaces, where numerous cells were situated on an electrode. This is in line with what is thought to happen in vivo^[^ ^39^, ^68^ ^]^ and is important to detect any changes in electrode–electrolyte interface that might be caused by this attachment. As these cells are seeded into a tissue‐engineered gel, the cells can also remodel and change this construct. In line with previous studies,^[^ ^42^, ^69^ ^]^ the cells cause significant contraction, ultimately resulting in contraction of the construct away from some electrodes that were originally embedded in construct at the beginning of the experiment. These electrodes show full recovery from an electrical perspective (data included in Figure S4, Supporting Information). This result is very promising for patients in that we also show electrodes can return to their original state, indicating that the development of treatments for the reduction or reversal of fibrosis has the potential to restore degradation in stimulation efficiency in clinical scenarios.

To design a new electrical marker of fibrosis progression, we first needed to understand the complex impedance changes over time in our model. We proposed a new electrical circuit to represent the changes in our model of cochlear fibrosis and showed significant changes in complex impedance over time. The elements representing the bulk of the construct (R_1_ and C) showed significant changes over time, while the CPE representing the electrode–electrolyte interface did not. This suggests that biological changes affecting electrical impedance can be explained by changes in the bulk of the construct, such as ECM formation and reorganization, rather than changes in the electrode‐electrolyte interface. In line with the complex impedance results, full voltage waveform recordings showed significant changes in the clinically measurable contact impedances over time, as well as in a newly proposed electrical marker, SPPR. The SPPR is directly measurable in patients and could allow for earlier detection of fibrosis formation and progression allowing for earlier treatment intervention. This marker, in addition to the further information we show to be retrievable from fitting full voltage waveforms, could also be utilized as a measurement tool in drug developing and testing studies.

EIS revealed changes in both impedance magnitude and phase angle over time and when modeled with our proposed circuit, revealed significant changes for those circuit elements representing the bulk of the construct. The changes in R_1_, however, are of a larger magnitude than the changes in C, suggesting absolute impedance magnitude changes are due to an increase in the resistance of the construct. No significant changes from day 2 to endpoint for the CPE representing the EE interface, even with cells visibly attached on the electrode surface, were found. A recent study by Fuentes‐Vélez et al. used the same electrical circuit as presented in the current study as a marker of liver fibrosis in mice.^[^ ^70^ ^]^ Liver fibrosis follows a wound‐healing response similar to what is thought to happen intracochlearly post‐implantation and includes an increase in ECM deposition.^[^ ^71^ ^]^ The authors saw an increase in bulk resistance, similar to that presented in the current study, when stimulating liver fibrosis and correlated this increase in resistance to the formation of ECM. This supports the use of our presented circuit model and suggests changes seen in this study could be due to cellularly mediated ECM alterations. Furthermore, our findings are in line with previous patient studies modeling fibrosis through voltage waveforms with the Tykocinski et al. circuit, where a change in access resistance is found over time.^[^ ^36^, ^56^, ^60^, ^62^ ^]^ We also tested this circuit model on our EIS data and found a large error for fitting across multiple frequencies, indicating that this model oversimplifies complex impedance. This has been previously described by Mesnildrey et al., who found a large residual error when using the simple RC circuit for the EE interface and proposed the use of a CPE instead for both EIS and voltage waveform fitting.^[^ ^72^ ^]^ Combining these observations, the circuit model presented in this study provides a more accurate picture of the electrical changes present during cochlear fibrosis formation. This model could potentially be used to study other types of input pulses, such as triphasic of pseudomonophasic pulses, for which SPPR could also be sensitive.

To allow for easily measurable data in CI patients using current clinical software and to provide a comparison with currently collected data from patients, we measured voltage waveform responses at each electrode. The clinically measurable contact impedances showed an increase over time, which is in line with studies examining contact impedances and fibrosis formation.^[^ ^4^, ^19^, ^35^, ^56^ ^]^ As mentioned above, we present a new electrical marker that would require only one extra timepoint to be measured and so provides an opportunity to easily expand data collection in patients. Interestingly, the inflection point for our SPPR marker was at an earlier timepoint than for the measured contact impedances. We were able to test our SPPR in patients at 2 timepoints postoperatively, which revealed a significant change on a group level in SPPR from 3 to 5 months postoperative while no significant change was found for contact impedances between these timepoints. However, interpretation of the statistical tests on this data should be done with caution as the sample size is small. Additionally, no control measure for fibrosis is present. To further test SPPR as a marker for fibrosis formation in patients, as well as test its correlation with residual hearing, a large patient study with intra‐operative and post‐operative timepoints of SPPR and auditory thresholds should be done. This would allow for a clearer indication of no fibrosis present (intra‐operatively) to fibrosis present (post‐operatively) than at only post‐operative timepoints as presented here.

We fitted our circuit model of fibrosis encapsulation to voltage waveforms measured and found significant correlations with the output of complex impedance fitting, showing an opportunity for additional information extraction from voltage waveforms in CI patients, which is possible with research software.^[^ ^38^, ^61^, ^73^ ^]^ However, proposed fitting of full voltage waveforms needs to be optimized further and needs to include a circuit model fitting to CI patients rather than an in vitro model. Our VW fitting had a large percentage of values capped at the limits and needed fixed values for the EE interface.

The model presented in this paper could be used as a drug‐testing platform, where changes in complex impedance, SPPR, and contraction could be used to test ways to inhibit or even reverse fibrosis. Patient biopsies could be used to build patient‐specific models of cochlear fibrosis. In our study, we did not observe any effects on our cells from applied electrical stimulation, despite contrary observations in some studies.^[^ ^74^ ^]^ Analysis of different stimulation regimens could yield different results, which would be easily achievable using our model. However, this criterion for examination was outside the range of our goals for this study.

As our system is meant to represent the immune response to CI implantation, we have notably not included immune cells within this model. Fibrosis in vivo is complex and involves other cell types beyond just fibroblasts.^[^ ^15^, ^16^, ^75^, ^76^ ^]^ Immune cells play a major role in the development and progression of fibrotic tissue. Previous animal studies indicate that after CI implantation, fibrin is first adsorbed onto electrodes. This matrix is infiltrated with macrophages and leukocytes, whose presence is reduced upon the infiltration of fibroblasts, which has been shown to occur around 7 days post‐implantation.^[^ ^12^, ^14^, ^15^ ^]^ Our model focuses on this latter stage of development after fibroblast infiltration. Of note, the presence of these immune cells plays a major role in the development of fibrotic response in vivo and would, therefore, likely have an effect on our model if present. We would speculate that the addition of immune cells would produce a more accurate timeline for fibrosis with possible changes in tissue morphology and structure. However, given that our model mostly focuses on the electrical response from the CI electrodes, these changes are unlikely to result in a different outcome from that which we observed.

One limitation of this model is that clinical fibrous encapsulation is attached to the walls of the cochlea, making longitudinal contraction to the levels seen in our study less likely.^[^ ^29^, ^77^ ^]^ This effect is also influenced by the positioning of the electrode array positioning in the tapered 3D structure of the cochlea, which could influence baseline complex impedance.^[^ ^65^ ^]^ On the next iterations of this model, we envision incorporating different characteristics of the cochlear environment using a tapered conical model of the cochlea. In this cochlea‐shaped bioreactor, we can incorporate testing of current spread towards the auditory nerve with fibrosis development to help understand CI performance changes due to bulk tissue formation. Nevertheless, this study shows significant and large changes from baseline in complex impedance and allowed us to present electrical changes in real‐time on a clinical electrode, which we were able to translate to a directly measurable electrical marker for fibrosis in CI patients.

## 4 Conclusion

In conclusion, this study presents a tissue‐engineered model of fibrosis progression on a clinical cochlear implant array. It demonstrates complex impedance as a marker of fibrosis progression and applies the changes found in complex impedance to directly measurable cochlear implant patient data. A new marker, the SPPR, provides a potential mechanism for gauging cochlear implant fibrosis formation progress in patients, with no additional software or equipment needed. These findings can be used to track fibrosis formation in patients in real‐time, allowing for earlier treatment intervention, and can be used in drug‐testing platforms to test and develop new treatments inhibiting fibrosis and therefore combating residual hearing loss. The findings in this study hold the potential for generalization to other neural implants with fibrosis formation, opening up new areas of exploration and treatment, for improving implant science.

## 5 Experimental Section

### Study Design

The overall goal of this study was to find an electrical biomarker for the development of fibrosis in a 3D bioreactor model with visual feedback in real‐time. The 3D bioreactor models (Figure 1A) contained a cochlear implant electrode array, identical to those used clinically, with fibroblasts 3D‐seeded into a fibrin gel covering the electrode array. The bioreactors were grouped in an unstimulated and stimulated groups, where malfunctioning electrode arrays were placed in the unstimulated group.

During six timepoints over a course of 14 days, electrochemical impedance spectroscopy (EIS) and full voltage waveform responses were measured for the stimulated group. Additionally, contraction status of the construct was captured with a hand‐held digital microscope at seven timepoints (including day 0). The experiments were repeated three times, with five bioreactors in the first round (*n* = 2 for stimulated, *n* = 3 for unstimulated), six in the second round (*n* = 2 for stimulated, *n* = 4 for unstimulated), and eight in the final round (*n* = 4 for stimulated, *n* = 4 for unstimulated). Prior to each experiment, the bioreactors were assigned to an assay for which tissue was collected on day 14. The assays included a Hoechst Deoxyribonucleic Acid (DNA) assay, histology and confocal imaging.

The experiments were ended on day 14 to prevent the construct from contracting off the electrode fully, at which point not enough tissue would be available for a follow‐up assay. Outliers that were present in the electrical dataset were excluded based on their complex impedance response (see EIS section for details).

For a detailed description of the experimental methods described in the results, see the Detailed Methods section in the Supporting Information.

### Bioreactor Setup

MED‐EL (Innsbruck, Austria) cochlear implant electrode arrays were manufactured for the purpose of this study. The electrode arrays are identical to the arrays of clinical implants within the MED‐EL FLEX series, consisting of 12 platinum‐iridium contacts in a silicone array (Figure 1B).^[^ ^78^ ^]^ The six basal contacts are always double‐sided, whereas the six apical electrodes are either single or double and when relevant, this is included in the statistical analysis (e.g., of contraction). A platinum‐iridium MED‐EL clover electrode was used as a ground electrode within the bioreactor (Figure 1A).^[^ ^79^ ^]^ To secure the wiring of the setup, several 3D‐printed holders were produced. These holders were designed in Autodesk Fusion 360 and printed on a CADworks3D M50‐405 printer (30 µm *x*–*y*‐resolution, CADworks3D, Toronto, Canada) using CADworks3D Master Mold resin. An opening was made in the lid of the conical in which a rubber gasket and a 3D‐printed holder was placed to include the leads of the electrode array and ground electrode (Figure 1A), as well as a sterile filter (0.22 µm, Merck Millex, Fisher Scientific).

Prior to placing the electrode array and ground within culture media in the conical, a fibrin gel was cast around the electrode array. A 3D‐printed platform was designed to keep the electrode array in place, while the construct was cast within a 3% w/v Pluronic‐coated (Pluronic F‐127, Sigma–Aldrich) silicone tube. The protocol for fibrin gel fabrication follows the Roberts et al. approach.^[^ ^42^ ^]^ The fibrin gel was produced by mixing a fibrinogen stock solution (bovine fibrinogen type I–S, Sigma Aldrich) in HEPES‐buffered saline (HBS, 20 mm HEPES (Sigma–Aldrich), 150 mm NaCl (Fisher Scientific), pH 7.4), telomerase‐immortalized fibroblasts (TIFs, a gift from Ellen Van Obberghen–Schilling, Institut de Bioligie de Valrose) and thrombin (bovine thrombin, Sigma–Aldrich) in CaCl_2_ (VWR international, 120 mm CaCl_2_ in HBS) to reach a final concentration of 12 mg mL^−1^ fibrinogen, 1 U mL^−1^ thrombin, 20 mm CaCl_2_ and 1500 cells µL^−1^.^[^ ^42^, ^80^ ^]^ The construct was allowed to react for 2 h at 37 °C and 5% CO_2_ before placing a stainless‐steel clip at the apical end of the electrode array, to act as a weight, and for the construct and ground electrode to be immersed in 10 mL of culture media within a conical. Culture media existed of Advanced Dulbecco's Modified Eagle Medium (DMEM, Gibco, ThermoFisher Scientific) supplemented with 20% Fetal Bovine Serum (FBS, Sigma–Aldrich), 2% HEPES (Gibco, ThermoFisher Scientific), 1% GlutaMAX (Gibco, ThermoFisher Scientific), 0.5% penicillin–streptomycin (10 000 U mL^−1^, Gibco, ThermoFisher Scientific) and 0.1% gentamicin (Sigma–Aldrich). On day 2 of the experiment, 10 ng mL^−1^ active recombinant human transforming growth factor *β*1 (TGF‐*β*1, Abcam) was added to the media of all bioreactors. Cell culture media was changed after data collection for each timepoint, leading to an incubation time of TGF‐*β*1 of 2 days.

### Contraction

To track localization of the construct relative to the individual electrodes, photos were taken at seven timepoints (on the day of fabrication and every measurement timepoint thereafter) using digital microscope (Dino‐Lite, Premier AM4113T). Whether the construct was on or off individual electrodes was decided by visual inspection and subsequent analysis (EIS and voltage waveform analysis) was grouped on this basis.

Contraction analysis was performed using ImageJ software. Image scaling was calibrated to the known mid‐to‐mid electrode contact spacing for each image. The mean and standard deviation of the calculated scaling was then used to calculate the length of the construct. Error bars in Figure 2B for the individual tracings are the standard deviation of the scaling. Reproducibility and repeatability of this method was tested with a Gage R&R study on 3 repeats of the same electrode array over time (Figure S1A, Supporting Information), using MATLAB's (R2020b) *gagerr* function.^[^ ^81^ ^]^ Relative contraction was calculated as the percentage contraction from the length of the construct on day 0. Images were stitched together for the purpose of Figure 2A using MosaicJ.^[^ ^82^ ^]^

### DNA Assay

DNA was quantified for a subset of constructs following the Kim et al. protocol for papain digestion of the constructs and Hoechst 33258 fluorescence assays.^[^ ^83^ ^]^ On day 14, the constructs were removed from the electrode array and kept at −80°C. Prior to digestion, the samples were lyophilized for 48 h at −60 °C and 0.2mBar (TelStar LyoQuest HT40). Samples were digested in a phosphate buffer with EDTA (PBE, 0.1 m Na_2_HPO_4_ (Sigma–Aldrich), 0.01 m Na_2_EDTA (Invitrogen, ThermoFisher), pH 6.5) based papain digest buffer (PDB) containing 0.1 m l‐Cysteine (Alfa Aesar, ThermoFisher) and 0.125 mg mL^−1^ Papain (Sigma–Aldrich). Samples were digested in 1 mL PDB for 12 h in a 60 °C water bath. Simultaneously, isolated genomic DNA from T‐47D cells (human breast cancer cell line, the DNA was a gift from Stephanie Mack, Cancer Research UK, University of Cambridge) was diluted with PDB to a concentration of 100 µg mL^−1^. Immediately following sample digestion, the assay was performed using Hoechst 33258 dye (0.1 µg mL^−1^ final concentration, Abcam) in a TES buffer (10 mm TrisBase (Sigma–Aldrich), 1 mm Na_2_EDTA, 0.1 mm NaCl (Sigma–Aldrich), pH 7.4). A serial dilution of DNA standard from 0 to 50 µg mL^−1^ was made and the samples were plated in triplicates. Fluorescence intensity was measured (TECAN Spark microplate reader) at an excitation wavelength of 348 nm and emission wavelength of 456 nm. Fluorescence was related to sample concentration using a linear regression model (*fitlm*, MATLAB).

### Histology

Samples for histology were prepped on day 14 by fixing the construct in 4% paraformaldehyde (PFA, Thermo Scientific) for 2 h. Thereafter, the construct was removed from the electrode array and cut in transversal and longitudinal slices (Figure 2C). The IMS Histopathology Core, MRC Metabolics Unit [MC_UU_00014/5], stained the slides for Haemotoxylin and Eosin (H&E), Ki‐67 and picrosirius red (PSR). Slides were visualized using an EVOS microscope (ThermoFisher). Collagen birefringence for PSR‐stained slides was visualized using Thorlabs LPVISE2×2 linear polarizers.

### Confocal Imaging

Samples (2 constructs, from Exp3, one stimulated, one unstimulated) for confocal imaging were fixed on day 14 in 4% PFA for 15 min. While keeping the construct on the electrode array, the construct was permeabilized for 10 min with 1% Triton‐X (Fisher Scientific) in phosphate‐buffered saline (PBS, Oxoid Ltd.). The constructs were first incubated with phalloidin‐iFluor 594 reagent (Abcam) for 1.5 h at a concentration of 4 µL mL^−1^ PBS. Subsequently, and after PBS washing, the constructs were incubated with Hoechst 33258 (Abcam) for 15 min. The constructs were then imaged using a confocal microscope (Axia Observer Z1 LSM 800, Zeiss) and images were extracted using ZEN software (ZEISS).

### Electrochemical Impedance Spectroscopy (EIS)

The electrode array and ground electrode were connected to an impedance analyzer (RS PRO LCR‐6100) via printed circuit boards and a data acquisition system (DAQ, USB‐6212, National Instruments), powered by a power supply (Keithley 2614B SourceMeter). An AC signal with an amplitude of 100 mV was used to collect complex impedances from 10 Hz to 100 kHz, with 10 frequencies per decade at equivalent logarithmic intervals. The order of stimulation of the electrodes on the array was randomized using LabVIEW (v2018, National Instruments Corp.) to a total of three repeats per electrode per timepoint.

The mean and standard deviation per three repeats were calculated for the absolute impedance magnitude and the corresponding phase angle in MATLAB. To exclude outliers (e.g., broken electrodes or an air bubble present on the electrode), timepoints for individual electrodes on the arrays were excluded when the 25% highest standard deviations of the phase angle (*θ* in °) were higher than 4°, the mean absolute impedance magnitude (|Z| in Ω) was higher than 50 kΩ at 10 Hz, or the mean phase angle was between −20 and 0° at any point from 10 Hz and 1 kHz. Any further analysis was done with the mean of the three repeats.

EIS data was then modeled using ZView (v3.1, Scribner Associates Inc.) to fit an equivalent circuit (Figure 4A), consisting of a constant phase element (CPE) representing the electrode‐electrolyte interface, a resistor (R_1_) in parallel with a capacitor (C) representing the bulk of the gul, and a resistor (R_2_) representing the resistive components of the media and the ground. Start values for the circuit element modeling were extracted from the mean absolute impedance magnitude at the first available timepoint. The start value of the constant phase of the CPE (CPE‐P) was always set at 0.8(*−90°). The magnitude of the CPE (CPE‐T) was then calculated by solving for *Y* at 10 Hz in the mathematical model of a CPE:

(1) $$Z_{CPE}=\frac{1}{Y\left(j\omega \right)\rho }$$

where *Y* is the magnitude of the CPE (CPE‐T), *p* is the chosen CPE‐P value and *ω* is the angular frequency defined as 2*πf*.^[^ ^84^ ^]^ The start value of both resistors (*R*_1_ and *R*_2_) was the absolute impedance magnitude at 100 kHz. The start value of the capacitor (C) was found by solving for C at 10 Hz in the mathematical model of a capacitor:

(2) $$Z_{C}=\;\frac{1}{j\omega C}$$

The data were then fitted using ZView's complex calc‐modulus data weighting fitting, with a maximum of 100 iterations and 100 optimization iterations. Circuit element *R*_2_ was not expected to change over time and was therefore fixed from the first available timepoint. The start values for following timepoints were the outcome of the previous timepoint.

Additionally, an example response was fitted with the simple circuit described for voltage waveform responses of cochlear implants (Figure S5A, Supporting Information).^[^ ^62^ ^]^ Start values for this circuit included identical approaches for the resistors and capacitors as described for the proposed circuit.

### Voltage Waveforms

In order to measure full voltage waveforms, as measured during clinical contact impedances, the electrode array was connected to a CI emulator (Advanced Bionics, AB, CA, USA). With this emulator, current stimuli can be generated by the Bionic Ear Data Collection System software (BEDCS, AB) when connected to a headpiece, an external sound processor (AB Clarion Platinum), a Clinician's Programming Interface (CPI‐2, AB‐6500, Clarion) and a laptop. The electrode array was stimulated with monopolar biphasic charge‐balanced pulses with a phase duration of 50 µsec and an amplitude of 50 µA, both cathodic‐leading and anodic‐leading. Recordings were made with an oscilloscope (Teledyne LeCroy HDO4054A‐MS, LeCroy Corp., New York, USA). The order of stimulation, and thus recording, were randomized using LabVIEW to a total of 15 repeats per pulse and electrode. When EIS recordings exhibited unusual high absolute impedances across all frequencies, the electrode was excluded from voltage waveform data collection for that timepoint.

The collected voltage waveforms were filtered using a first order digital Butterworth low‐pass filter with a cut‐off frequency of 80 kHz, using MATLAB's function *butter* and *filter*. For easier handling of data, the sample rate of the waveforms was then decreased by a factor 20 using MATLAB's *downsample*, to a final sampling frequency of approximately 6 MHz. A small offset, which was due to environmental noise, was corrected by normalizing the downsampled waveform to the first 500 datapoints/80 µs in which no stimulus was applied. The mean and standard deviation across 15 repeats was then calculated in MATLAB.

Clinically measurable contact impedances were calculated by taking the peak of the first phase of the cathodic‐leading biphasic pulse and dividing it by the input current (50 µA). Additionally, the SPPR was calculated (Figure 5C) as the second phase voltage peak as a percentage of the first phase voltage peak. Both measures were also presented normalized to the value at day 2.

To correlate the second phase voltage peak ratio with EIS‐fitted circuit elements, a linear function was fitted to the SPPR over time using MATLAB's *polyfit* and *polyval*. Only timepoints with construct on the electrode, with a minimum of three available timepoints and timepoints beyond day 7 (inflection point of R_1_ in Figure 4C) were included.

### Modelling and Simulation of Voltage Waveforms

After modeling the EIS circuits to the data, voltage waveforms were simulated with the outputs of the model in a similar fashion to Jiang et al.^[^ ^85^ ^]^ The Laplace transform of a biphasic current pulse can be expressed as:

(3) $$I\;=\;-\;\frac{i_{amp}}{s}\left(1-2e^{-sT_{0}}+\;e^{-2sT_{0}}\right)$$

where *i_amp_* is the current amplitude of the pulse and *T_0_* is the phase duration. The total impedance of the circuit (Figure 4A) can be expressed as:

(4) $$Z_{total}=\;\frac{1}{Ys^{p}}+\;\frac{R_{1}}{1\;+\;sR_{1}C}+R_{2}$$

Since Ohm's law states the voltage is the product of current and impedance, the voltage can be expressed as:

(5) $$V=\;-\;\frac{i_{amp}}{s}\left(1-2e^{-sT_{0}}+\;e^{-2sT_{0}}\right)\cdot \left(\frac{1}{Ys^{p}}+\;\frac{R_{1}}{1+sR_{1}C}+\;R_{2}\right)$$

To obtain the voltage waveform response in the time‐domain, an inverse Laplace transform was performed on the formula for voltage in the frequency‐domain:

(6) $$\begin{array}{ccc} L^{-1}\;\left\{V\right\} & = & \;-\;i_{amp}L^{-1}\left\{\frac{1}{s}\left(\frac{1}{Ys^{p}}+\;\frac{R_{1}}{1+sR_{1}C}+\;R_{2}\right)\right\}\\ & & \cdot \left\{\delta \left(t\right)-2\delta \left(t-T_{0}\right)+\delta \left(t-2T_{0}\right)\right\} \end{array}$$

This can be solved so that the voltage (*V*) in the time‐domain can be expressed as:

(7) $$V=-\;i_{amp}\left\{\begin{array}{c} \left(\frac{t^{p}}{Y\Gamma \left(p+1\right)}+R_{1}\left(1-e^{-\;\frac{t}{R_{1}C}}\right)\;+\;R_{2}\right)\cdot u\left(t\right)-\;2\cdot \left(\frac{{\left(t-T_{0}\right)}^{p}}{Y\Gamma \left(p+1\right)}+R_{1}\left(1-e^{-\;\frac{t-T_{0}}{R_{1}C}}\right)\;+\;R_{2}\right)\cdot u\left(t-T_{0}\right)\\ +\left(\frac{{\left(t-2T_{0}\right)}^{p}}{Y\Gamma \left(p+1\right)}+R_{1}\left(1-e^{-\;\frac{t-2T_{0}}{R_{1}C}}\right)\;+\;R_{2}\right)\cdot u\left(t-2T_{0}\right) \end{array}\right\}$$

where *u(t,T_0_)* is a step function. The results of the EIS modelling within ZView® could then be used to simulate full voltage waveforms (Figure 6A). Additionally, this formula was then used to find the circuit element sizes when using voltage waveforms as an input.

To find the circuit elements, the normalized, filtered, mean voltage waveform of 15 repeats was used as an input. All voltage waveforms were fitted for which a comparative EIS fitting was available on day 2. First, the step function *u(t,T_0_)* was set to align with the input voltage waveform by finding the start of the first phase using MATLAB's *findchangepts* function, finding changes in root‐mean‐square level of the waveform. Next, MATLAB's *fmincon* was used to find the lowest sum‐of‐squares output for alternating values of the circuit elements. CPE‐P (*p* in formula), CPE‐T (*Y* in formula), and *R*_2_ were fixed as the EIS‐fitted value on day 2. For *R*_1_ and *C*, wide limits were set as 50–5000 Ω for *R*_1_ and 10^−13^–10^−7^ F for *C*. The fitting process was repeated for all unique combinations of 30 linearly spaced values of *R*_1_ and *C*, within their set limits for each waveform. Therefore, the fitting process was repeated 900 times per waveform and the solutions for *R*_1_ and *C* corresponding to the lowest sum‐of‐squares fit were chosen as the output (see Figure S7E, Supporting Information, for distribution of final sum‐of‐squares). For the purpose of the correlation with EIS elements in Figure 6D, output values capped at the limits for one of the circuit elements were excluded (see Figure S7C, Supporting Information).

### Patient Data

Data were collected from four patients implanted with a Cochlear Ltd. (Sydney, Australia) using Custom Sound EP 6.0, which allows for data collection of voltage per unit input current at several timepoints within the waveform. Three patients (P1, P3, P4) received a later‐wall electrode (C622) while one patient (P2) received a modiolus‐hugging electrode (CI612). One timepoint was collected intraoperatively, while four timepoints were collected 3 months and 5 months postoperatively (Figure 7A). The implanted headpiece was connected to a CP910 research sound processor equipped with an CP1000 coil and diametric magnet that was directly connected to a programming POD and testing laptop. The input pulse is a cathodic‐leading charge‐balanced biphasic pulse, phase duration of 25 µs and interphase gap of 9 µs. Contact impedances were calculated as done clinically at the end of the first phase (25 µs). The current amplitude for this pulse post‐operatively was 125 µA for all patients. The input amplitude for the intraoperatively measured contact “impedance” was varied by the Custom Sound EP 6.0 software adaptively based on conditioning measures and was within the range of 435–1055 µA. Contact “impedances” were calculated as done clinically at the end of the first phase (25 µs) and are normalized to their input current. Since it was not possible to measure the exact end of the second phase with clinical software, the second phase peak ratio was calculated using timepoints 6 µs into each phase. Current levels required to achieve mean comfortable loudness (MCL) were determined behaviorally using evoked compound action potential (ECAP) stimulation patterns at 80 pulses s^−1^ (pps) of biphasic current pulses with 37 µs (P1, P2, P4) or 50 µs (P3) phase durations and 8 µsec interphase gaps. MCLs are expressed in the log‐scale unit of current levels (CL), where current in µA is equal to 17.5 × (100^(CL/255)^).

### Statistical Analysis

All data are shown in the figures as mean ± standard deviation unless otherwise stated. Statistical analysis was performed using MATLAB (vR2020b) and IBM SPSS Statistics (v28). MATLAB's functions *mean*, *geomean, std, anovan, multcompare, corrcoef, fitlm*, and *aoctool* were used. Statistical significance was set at *α* = 0.05 unless otherwise stated. *p*‐Values and *n*‐values were reported on throughout. For Pearson's correlation coefficient (*r*), the 95% confidence interval was reported on.

## Conflict of Interest

The authors declare no conflict of interest.

## Supporting information

Supporting Information

## Data Availability

The data that support the findings of this study are available on request from the corresponding author. The data are not publicly available due to privacy or ethical restrictions.

## References

[1] W. H. Organization , World Report on Hearing, World Health Organization, Geneva 2021.

[2] J. M. Gaylor , G. Raman , M. Chung , J. Lee , M. Rao , J. Lau , D. S. Poe , JAMA Otolaryngol. Head Neck Surg. 2013, 139, 265. 23429927 10.1001/jamaoto.2013.1744

[3] B. S. Wilson , M. F. Dorman , J. Rehabil. Res. Dev. 2008, 45, 695. 18816422 10.1682/jrrd.2007.10.0173

[4] E. Bas , J. Bohorquez , S. Goncalves , E. Perez , C. T. Dinh , C. Garnham , R. Hessler , A. A. Eshraghi , T. R. Van De Water , Hear Res. 2016, 337, 12. 26892906 10.1016/j.heares.2016.02.003

[5] M. Wilk , R. Hessler , K. Mugridge , C. Jolly , M. Fehr , T. Lenarz , V. Scheper , PLoS One 2016, 11, e0147552. 26840740 10.1371/journal.pone.0147552 PMC4739581

[6] R. Klopfleisch , F. Jung , J. Biomed. Mater. Res. 2017, 105, 927. 10.1002/jbm.a.35958 27813288

[7] N. Noskovicova , R. Schuster , S. van Putten , M. Ezzo , A. Koehler , S. Boo , N. M. Coelho , D. Griggs , P. Ruminski , C. A. McCulloch , B. Hinz , Nat. Biomed. Eng. 2021, 5, 1437. 34031559 10.1038/s41551-021-00722-z

[8] M. Gulino , D. Kim , S. Pané , S. D. Santos , A. P. Pêgo , Front. Neurosci. 2019, 13, 689. 31333407 10.3389/fnins.2019.00689 PMC6624471

[9] M. J. Foggia , R. V. Quevedo , M. R. Hansen , Laryngoscope Investig. Otolaryngol. 2019, 4, 678. 10.1002/lio2.329 PMC6929576 31890888

[10] R. Chen , A. Canales , P. Anikeeva , Nat. Rev. Mater. 2017, 2, 16093. 31448131 10.1038/natrevmats.2016.93 PMC6707077

[11] A. Carnicer‐Lombarte , S.‐T. Chen , G. G. Malliaras , D. G. Barone , Front. Bioeng. Biotechnol. 2021, 9, 622524. 33937212 10.3389/fbioe.2021.622524 PMC8081831

[12] M. T. Rahman , D. A. Chari , G. Ishiyama , I. Lopez , A. M. Quesnel , A. Ishiyama , J. B. Nadol , M. R. Hansen , Hear Res. 2022, 422, 108536. 35709579 10.1016/j.heares.2022.108536 PMC9684357

[13] A. P. Sanderson , E. T. F. Rogers , C. A. Verschuur , T. A. Newman , Front. Neurosci. 2019, 12, 1048. 30697145 10.3389/fnins.2018.01048 PMC6340939

[14] J. K. Choong , A. J. Hampson , K. M. Brody , J. Lo , C. W. Bester , A. W. Gummer , N. P. Reynolds , S. J. O'Leary , Hear Res 2020, 385, 107846. 31786442 10.1016/j.heares.2019.107846

[15] G. E. Kel , J. Tan , H. T. Eastwood , S. Wongprasartsuk , S. J. O'Leary , Otol. Neurotol. 2013, 34, 1595. 23928509 10.1097/MAO.0b013e31828f4929

[16] J. H. W. Distler , A.‐H. Györfi , M. Ramanujam , M. L. Whitfield , M. Königshoff , R. Lafyatis , Nat Rev Rheumatol 2019, 15, 705. 31712723 10.1038/s41584-019-0322-7

[17] C. Bester , T. Razmovski , A. Collins , O. Mejia , S. Foghsgaard , A. Mitchell‐Innes , C. Shaul , L. Campbell , H. Eastwood , S. O'Leary , Sci. Rep. 2020, 10, 2777. 32066743 10.1038/s41598-019-56253-w PMC7026160

[18] F. Heutink , T. M. Klabbers , W. J. Huinck , F. Lucev , W. J. van der Woude , E. A. M. Mylanus , B. M. Verbist , Radiology 2022, 302, 605. 34874202 10.1148/radiol.211400

[19] R. Ishai , B. S. Herrmann , J. B. Nadol , A. M. Quesnel , Hear Res 2017, 348, 44. 28216124 10.1016/j.heares.2017.02.012 PMC5738657

[20] R. M. Knoll , D. R. Trakimas , M. J. Wu , R. J. Lubner , J. B. Nadol , A. Ishiyama , F. Santos , D. H. Jung , A. K. Remenschneider , E. D. Kozin , Otol. Neurotol. 2022, 43, e153. 35015749 10.1097/MAO.0000000000003402

[21] M. A. Somdas , P. M. M. C. Li , D. M. Whiten , D. K. Eddington , J. B. Nadol, Jr. , Audiol. Neurotol. 2007, 12, 277. 10.1159/000103208 17536196

[22] A. Danielian , G. Ishiyama , I. A. Lopez , A. Ishiyama , Otol. Neurotol. 2021. 10.1097/MAO.0000000000003106 PMC8282738 33710156

[23] T. Kamakura , J. B. Nadol , Hear Res 2016, 339, 132. 27371868 10.1016/j.heares.2016.06.015 PMC5018452

[24] A. A. Eshraghi , C. Gupta , T. R. Van De Water , J. E. Bohorquez , C. Garnham , E. Bas , V. M. Talamo , Laryngoscope 2013, 123, S1. 10.1002/lary.23902 23382052

[25] J. Xu , R. K. Shepherd , R. E. Millard , G. M. Clark , Hear Res 1997, 105, 1. 9083801 10.1016/s0378-5955(96)00193-1

[26] C.‐H. Choi , J. S. Oghalai , Hear Res 2005, 205, 193. 15953528 10.1016/j.heares.2005.03.018 PMC3623675

[27] D. Chen , Y. Luo , J. Pan , A. Chen , D. Ma , M. Xu , J. Tang , H. Zhang , Front Cell Dev Biol 2021, 9, 740576. 34778254 10.3389/fcell.2021.740576 PMC8589109

[28] Y. Liu , C. Jolly , S. Braun , T. Janssen , E. Scherer , J. Steinhoff , H. Ebenhoch , A. Lohner , T. Stark , J. Kiefer , Hear Res 2015, 327, 89. 25987502 10.1016/j.heares.2015.04.019

[29] A. M. Quesnel , H. H. Nakajima , J. J. Rosowski , M. R. Hansen , B. J. Gantz , J. B. Nadol , Hear Res 2016, 333, 225. 26341474 10.1016/j.heares.2015.08.018 PMC4775460

[30] R. Briggs , S. O. ’Leary , C. Birman , K. Plant , R. English , P. Dawson , F. Risi , J. Gavrilis , K. Needham , R. Cowan , Hear Res 2020, 390, 107924. 32143111 10.1016/j.heares.2020.107924

[31] G. Paasche , F. Bockel , C. Tasche , A. Lesinski‐Schiedat , T. Lenarz , Otol Neurotol 2006, 27, 639. 16868511 10.1097/01.mao.0000227662.88840.61

[32] V. Scheper , R. Hessler , M. Hütten , M. Wilk , C. Jolly , T. Lenarz , G. Paasche , PLoS One 2017, 12, e0183820. 28859106 10.1371/journal.pone.0183820 PMC5578571

[33] Z. He , Y. Ding , Y. Mu , X. Xu , W. Kong , R. Chai , X. Chen , Front Cell Dev Biol 2021, 9, 730042. 34746126 10.3389/fcell.2021.730042 PMC8567027

[34] E. Ajay , N. Gunewardene , R. Richardson , Expert Opin. Biol. Ther. 2022, 22, 689. 35485229 10.1080/14712598.2022.2072208

[35] C. Newbold , S. Mergen , R. Richardson , P. Seligman , R. Millard , R. Cowan , R. Shepherd , Cochlear Implants Int 2014, 15, 191. 23998484 10.1179/1754762813Y.0000000050

[36] C. Newbold , R. Richardson , C. Q. Huang , D. Milojevic , R. Cowan , R. Shepherd , J. Neural Eng. 2004, 1, 218. 15876642 10.1088/1741-2560/1/4/005

[37] J. Choi , M. R. Payne , L. J. Campbell , C. W. Bester , C. Newbold , H. Eastwood , S. J. O'Leary , Otol. Neurotol. 2017, 38, 1433. 29135865 10.1097/MAO.0000000000001589

[38] M. Parreño , F. A. Di Lella , F. Fernandez , C. M. Boccio , S. A. Ausili , Front Digit Health 2020, 2, 582562. 34713054 10.3389/fdgth.2020.582562 PMC8521944

[39] C. Newbold , R. Richardson , R. Millard , C. Huang , D. Milojevic , R. Shepherd , R. Cowan , J. Neural Eng. 2010, 7, 056011. 20841637 10.1088/1741-2560/7/5/056011 PMC3543851

[40] D. L. Matera , W. Y. Wang , B. M. Baker , Nat. Rev. Mater. 2021, 6, 192.

[41] A. J. Boys , R. M. Owens , APL Mater. 2021, 9, 040903.

[42] I. V. Roberts , R. Donno , F. Galli , C. Y. L. Valdivieso , A. Siani , G. Cossu , A. Tirella , N. Tirelli , Mater. Sci. Eng., C 2022, 133, 112661. 10.1016/j.msec.2022.112661 35067436

[43] J. C. Deddens , A. H. Sadeghi , J. Hjortnaes , L. W. van Laake , M. Buijsrogge , P. A. Doevendans , A. Khademhosseini , J. P. G. Sluijter , Adv. Healthcare Mater. 2017, 6, 1600571. 10.1002/adhm.201600571 27906521

[44] D. N. Rocha , J. P. Ferraz‐Nogueira , C. C. Barrias , J. B. Relvas , A. P. Pêgo , Front. Cell Neurosci. 2015, 9, 377. 26483632 10.3389/fncel.2015.00377 PMC4586948

[45] A. A. Pathiraja , R. A. Weerakkody , A. C. von Roon , P. Ziprin , R. Bayford , J. Transl. Med. 2020, 18, 227. 32513179 10.1186/s12967-020-02395-9 PMC7282098

[46] V. S. Teixeira , V. Labitzky , U. Schumacher , W. Krautschneider , Curr. Directions Biomed. Eng. 2020, 6, 341.

[47] I. Giaever , C. R. Keese , Nature 1993, 366, 591. 8255299 10.1038/366591a0

[48] I. V. Roberts , D. Bukhary , C. Y. L. Valdivieso , N. Tirelli , Macromol. Biosci. 2020, 20, 1900283. 10.1002/mabi.201900283 31769933

[49] A. Balabiyev , N. P. Podolnikova , J. A. Kilbourne , D. P. Baluch , D. Lowry , A. Zare , R. Ros , M. J. Flick , T. P. Ugarova , Biomaterials 2021, 277, 121087. 34478933 10.1016/j.biomaterials.2021.121087 PMC8516434

[50] A. Montero , S. Acosta , R. Hernández , C. Elvira , J. L. Jorcano , D. Velasco , J. Biomed. Mater. Res., Part A 2021, 109, 500. 10.1002/jbm.a.37033 32506782

[51] T.‐L. Tuan , A. Song , S. Chang , S. Younai , M. E. Nimni , Exp. Cell Res. 1996, 223, 127. 8635484 10.1006/excr.1996.0065

[52] R. Lattouf , R. Younes , D. Lutomski , N. Naaman , G. Godeau , K. Senni , S. Changotade , J. Histochem. Cytochem. 2014, 62, 751. 25023614 10.1369/0022155414545787

[53] A. J. Boys , J. A. M. R. Kunitake , C. R. Henak , I. Cohen , L. A. Estroff , L. J. Bonassar , ACS Appl. Mater. Interfaces 2019, 11, 26559. 31267742 10.1021/acsami.9b03595 PMC6680087

[54] J. Kim , A. J. Boys , L. A. Estroff , L. J. Bonassar , ACS Biomater. Sci. Eng. 2021, 7, 1608. 33606521 10.1021/acsbiomaterials.0c01791

[55] T. Scholzen , J. Gerdes , J. Cell. Physiol. 2000, 182, 311. 10653597 10.1002/(SICI)1097-4652(200003)182:3<311::AID-JCP1>3.0.CO;2-9

[56] C. Newbold , R. Richardson , R. Millard , P. Seligman , R. Cowan , R. Shepherd , J. Neural Eng. 2011, 8, 036029. 21572219 10.1088/1741-2560/8/3/036029 PMC3147028

[57] U. A. Aregueta‐Robles , Y. L. Enke , P. M. Carter , R. A. Green , L. A. Poole‐Warren , IEEE Trans. Biomed. Eng. 2020, 67, 3510. 32340929 10.1109/TBME.2020.2989754

[58] S. F. Alhabib , Y. Abdelsamad , M. Yousef , F. Alzhrani , Eur. Arch. Otorhinolaryngol. 2021, 278, 3211. 32979117 10.1007/s00405-020-06382-0

[59] W. Sunwoo , H. W. Jeon , B. Y. Choi , Sci. Rep. 2021, 11, 22809. 34815432 10.1038/s41598-021-01862-7 PMC8611070

[60] V. D. Tejani , H. Yang , J.‐S. Kim , H. Hernandez , J. J. Oleson , M. R. Hansen , B. J. Gantz , P. J. Abbas , C. J. Brown , J. Assoc. Res. Otolaryngol. 2022, 23, 95. 34686938 10.1007/s10162-021-00809-z PMC8782980

[61] F. A. Di Lella , D. De Marco , F. Fernández , M. Parreño , C. M. Boccio , Otol. Neurotol. 2019, 40, S18. 31225818 10.1097/MAO.0000000000002214

[62] M. Tykocinski , L. T. Cohen , R. S. Cowan , Otol Neurotol 2005, 26, 948. 16151342 10.1097/01.mao.0000185056.99888.f3

[63] Y. Dong , J. J. Briaire , M. Siebrecht , H. C. Stronks , J. H. M. Frijns , Ear Hear. 2021, 42, 1397. 33974777 10.1097/AUD.0000000000001033 PMC8378542

[64] S. R. de Rijk , Y. C. Tam , R. P. Carlyon , M. L. Bance , Ear Hear. 2020. 10.1097/AUD.0000000000000837 PMC7115972 31923041

[65] C. K. Giardina , E. S. Krause , K. Koka , D. C. Fitzpatrick , IEEE Trans. Biomed. Eng. 2018, 65, 327. 29346102 10.1109/TBME.2017.2764881 PMC5929978

[66] T. L. Bruns , K. E. Riojas , R. F. Labadie , R. J. Webster Iii , IEEE Trans. Biomed. Eng. 2022, 69, 718. 34379586 10.1109/TBME.2021.3104104 PMC8918040

[67] L. Gärtner , A. Büchner , A. Illg , T. Lenarz , Laryngoscope 2021, 131, E1275. 33237572 10.1002/lary.29023

[68] L. Tang , J. W. Eaton , Mol Med 1999, 5, 351. 10415159 PMC2230429

[69] T. Zhang , J. H. Day , X. Su , A. G. Guadarrama , N. K. Sandbo , S. Esnault , L. C. Denlinger , E. Berthier , A. B. Theberge , Front. Bioeng. Biotechnol. 2019, 7, 196. 31475142 10.3389/fbioe.2019.00196 PMC6702460

[70] S. Fuentes‐Vélez , S. Fagoonee , A. Sanginario , M. Pizzi , F. Altruda , D. Demarchi , Biosensors 2022, 12, 116. 35200376 10.3390/bios12020116 PMC8869865

[71] G. P. Caviglia , C. Rosso , S. Fagoonee , G. M. Saracco , R. Pellicano , Panminerva Med. 2017, 59, 320. 28880053 10.23736/S0031-0808.17.03359-6

[72] Q. Mesnildrey , O. Macherey , P. Herzog , F. Venail , J. Neural Eng. 2019, 16, 016023. 30523898 10.1088/1741-2552/aaecff

[73] F. A. Di Lella , M. Parreño , F. Fernandez , C. M. Boccio , S. A. Ausili , Front Bioeng Biotechnol 2020, 8, 568690. 33072726 10.3389/fbioe.2020.568690 PMC7530401

[74] C. Chen , X. Bai , Y. Ding , I.‐S. Lee , Biomater. Res. 2019, 23, 25. 31844552 10.1186/s40824-019-0176-8 PMC6896676

[75] H. Jia , F. François , J. Bourien , M. Eybalin , R. V. Lloyd , T. R. Van De Water , J.‐L. Puel , F. Venail , Neuroscience 2016, 316, 261. 26718602 10.1016/j.neuroscience.2015.12.031

[76] C. E. Witherel , D. Abebayehu , T. H. Barker , K. L. Spiller , Adv. Healthcare Mater. 2019, 1801451. 10.1002/adhm.201801451 PMC6415913 30658015

[77] J. N. Fayad , A. O. Makarem , F. H. Linthicum , Otolaryngol Head Neck Surg 2009, 141, 247. 19643260 10.1016/j.otohns.2009.03.031 PMC2779735

[78] MED‐EL , MED‐EL: Electrode Arrays .

[79] M. M. Medina , R. Polo , E. Amilibia , F. Roca‐Ribas , M. Díaz , M. Pérez , A. Muriel , J. Gavilán , I. Cobeta , L. Lassaletta , Ear & Hearing 2020, 41, 1648. https://s3.medel.com/pdf/21617.pdf 33136639 10.1097/AUD.0000000000000883

[80] J. Munro , K. Steeghs , V. Morrison , H. Ireland , E. K. Parkinson , Oncogene 2001, 20, 3541. 11429701 10.1038/sj.onc.1204460

[81] R. K. Burdick , C. M. Borror , D. C. Montgomery , Soc. Ind. Appl. Math.: Am. Stat. Assoc. 2005.

[82] P. Thévenaz , M. Unser , Microsc. Res. Tech. 2007, 70, 135. 17133410 10.1002/jemt.20393

[83] Y.‐J. Kim , R. L. Y. Sah , J.‐Y. H. Doong , A. J. Grodzinsky , Anal. Biochem. 1988, 174, 168. 2464289 10.1016/0003-2697(88)90532-5

[84] M. Boillot , S. Didierjean , F. Lapicque , J. Appl. Electrochem. 2004, 34, 1191.

[85] C. Jiang , S. R. de Rijk , G. G. Malliaras , M. L. Bance , APL Mater. 2020, 8, 091102. 32929397 10.1063/5.0012514 PMC7470452

