# Supplementary material for: Tissue‐Engineered Cochlear Fibrosis Model Links Complex Impedance to Fibrosis Formation for Cochlear Implant Patients
Source: Adv Healthc Mater. 2023 Jun 22;12(24):2300732. doi: 10.1002/adhm.202300732 (PMC11468547; doi:10.1002/adhm.202300732)
Supplement: Supplementary file 1 — Supporting Information [file ADHM-12-2300732-s001.pdf]

ADVANCED HEALTHCARE MATERIALS

Supporting Information

for *Adv. Healthcare Mater.*, DOI 10.1002/adhm.202300732

Tissue-Engineered Cochlear Fibrosis Model Links Complex Impedance to Fibrosis Formation for Cochlear Implant Patients

*Simone R. de Rijk, Alexander J. Boys, Iwan V. Roberts, Chen Jiang, Charlotte Garcia, Róisín M. Owens and Manohar Bance**

Supporting Information

Tissue-engineered cochlear fibrosis model links complex impedance to fibrosis formation for cochlear implant patients

*Simone R. de Rijk, Alexander J. Boys, Iwan V. Roberts, Chen Jiang, Charlotte Garcia, Roisin M. Owens, Manohar Bance**

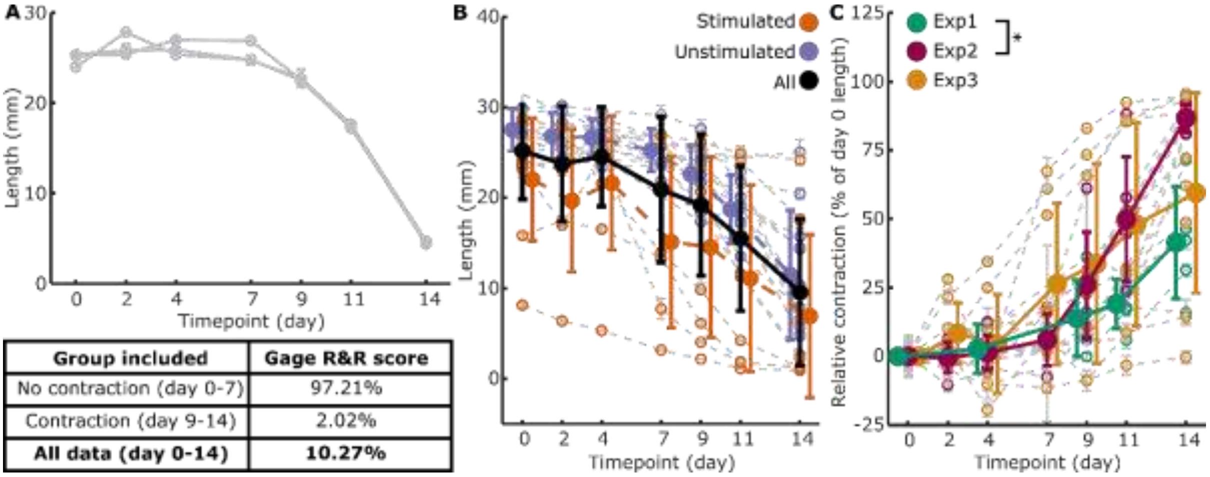

Group included	Gage R&R score
No contraction (day 0-7)	97.21%
Contraction (day 9-14)	2.02%
**All data (day 0-14)**	**10.27%**

Figure S1. Contraction analysis of the fibrin gels: method, absolute length and relative length grouped by experiment number. **(A)** Gage repeatability & reproducibility results on one dataset of contraction over time. Showing absolute length of the 3 repeats in the top figure and Gage repeatability & reproducibility scores in the table below, revealing high reliability of this method especially at the timepoints of interest after the gel started contracting. **(B)** Absolute length of the gel grouped in a stimulated, unstimulated and all group. Single datapoints are shown in grey lines with open circles. Mean ± standard deviation are shown for the stimulated and unstimulated groups and total. **(C)** Relative contraction of the gels, normalized to day 0 absolute length, over time, grouped per experiment number. Single datapoints are shown in grey lines with open circles. Mean ± standard deviation are shown for the 3 different experiments (Exp1 vs. Exp2, *p<0.05, univariate n-way ANOVA, Tukey's post-hoc test).

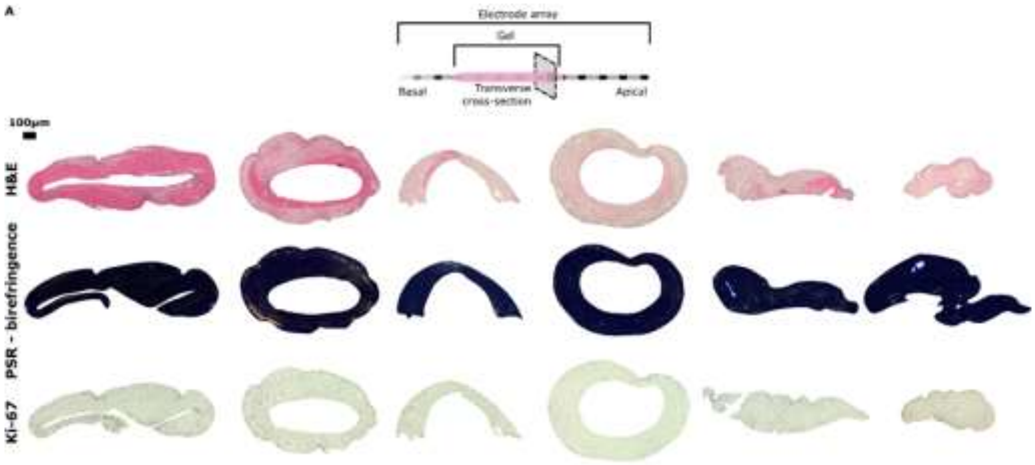

Figure S2. Histology stained with H&E, PSR and Ki-67. **(A)** Transverse cross-sections.

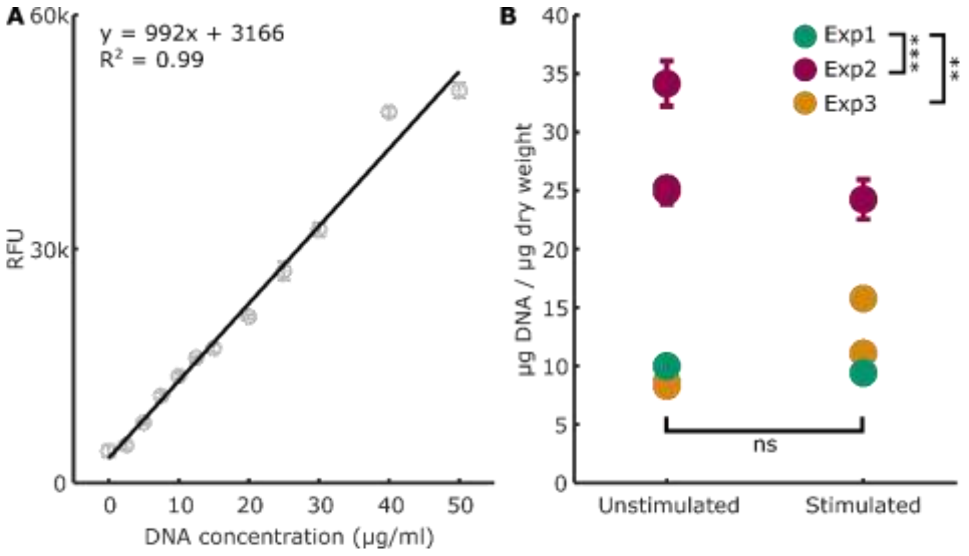

Figure S3. DNA concentrations as determined by a Hoechst 33258 fluorescence assay. **(A)** Ladder of serial dilutions of DNA standard, expressed in RFU (relative fluorescence units). Mean and standard deviation of triplicates are shown in grey, while the bold line shows the fit with a linear regression model. **(B)** DNA concentrations for different samples grouped by experiments (Exp1 vs. Exp2 ***p<0.005, Exp1 vs. Exp3 **p<0.01, univariate n-way ANOVA, Tukey's post-hoc test) and stimulation (ns = not significant, univariate n-way ANOVA).

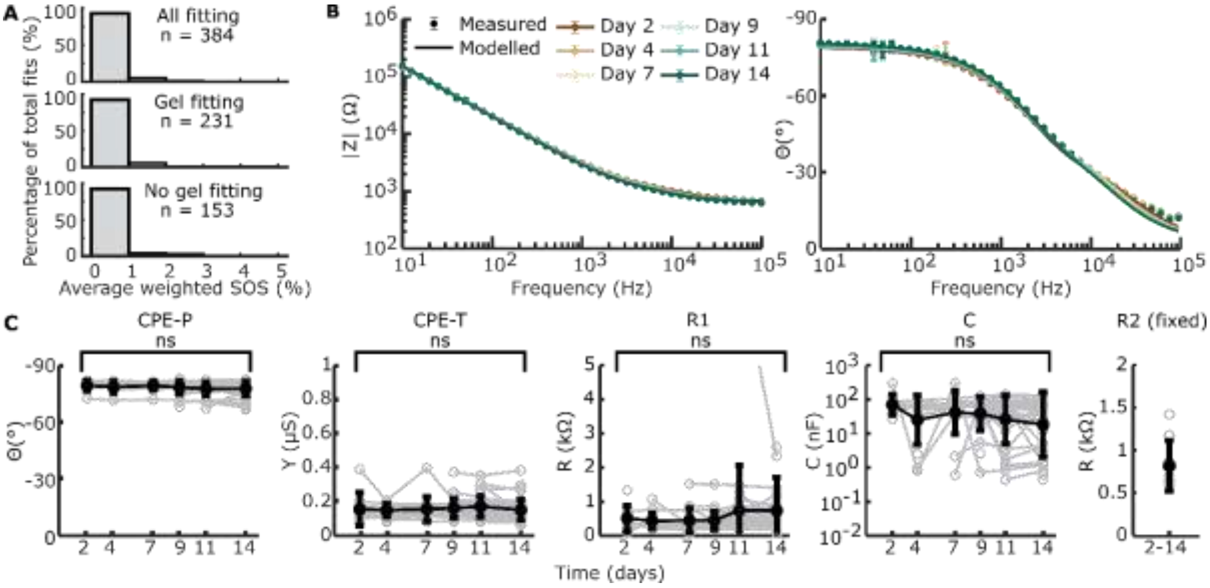

Figure S4. Complex impedance measured with electrochemical impedance spectroscopy (EIS) error of fit and results without gel on electrode. **(A)** Average weighted sum-of-squares, proportional to the average percent error, shown for all fittings, electrodes with gel encapsulation (gel fitting) and without gel on the electrode (no gel fitting). **(B)** Absolute impedance magnitude and phase angle of an example electrode over time without gel on the electrode, showing both measured and modelled values. Measured data is shown as mean ± standard deviation. **(C)** Modelled circuit elements over time of all timepoints and electrodes without gel on the electrode. Individual data is shown in grey. The arithmetic mean ± standard deviation is shown in bold black, except for C where the geometric mean and standard deviation is shown. All circuit elements show no significant (ns) changes from day 2 to day 14 (univariate n-way ANOVA, Tukey's post-hoc test).

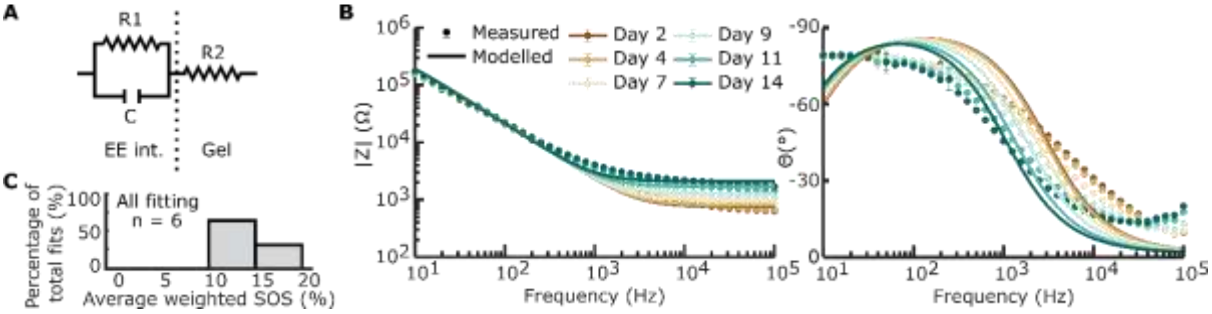

Figure S5. Complex impedance as measured with electrochemical impedance spectroscopy (EIS) modelled with a simple circuit. **(A)** Schematic of simple circuit with a resistor (R_1) in parallel with a capacitor (C) representing the electrode-electrolyte (EE) interface and an additional resistor (R_2) representing the bulk of the gel. **(B)** Absolute impedance magnitude and phase angle of an example electrode over time (same electrode as Fig. 4B, 5A), showing both measured and modelled values. Measured data is shown as mean ± standard deviation. **(C)** Average weighted sum-of-squares, proportional to the average percentage error, shown for the example electrode shown in (B).

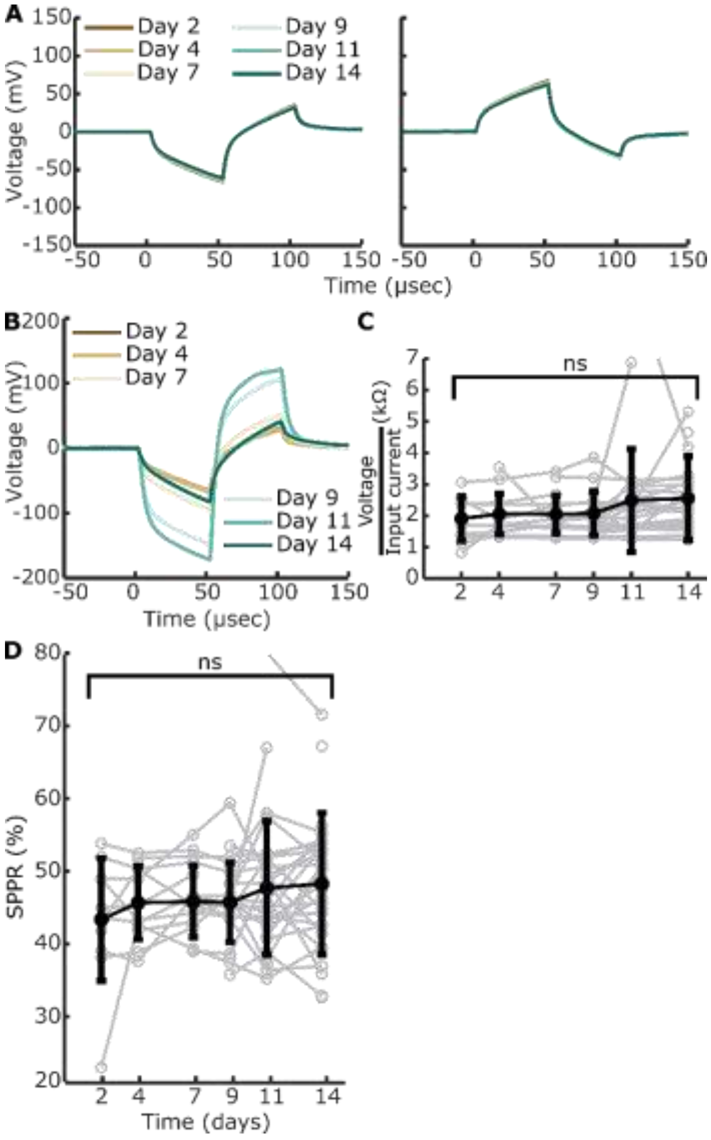

Figure S6. Measured voltage waveforms, contact 'impedances' and second phase peak ratio (SPPR) without gel on the electrode. **(A)** Example mean voltage waveforms over time for the same electrode as in fig. S4B, with a cathodic-leading biphasic pulse and anodic-leading biphasic pulse as an input. **(B)** Example mean voltage waveforms over time where the gel contracts off the electrode between day 11 and 14. **(C)** Absolute contact 'impedances' over time when there is no gel on the electrode. Individual traces are shown in grey, while mean ± standard deviation is shown in bold black. Absolute contact 'impedances' were not significantly different from day 2 to day 14 (ns = not significant, univariate n-way ANOVA, Tukey's post-hoc test). **(D)** Absolute SPPR (second phase peak ratio) over time when there is no gel on the electrode. Individual traces are shown in grey, while mean ± standard deviation is shown in bold black. Absolute SPPR were not significantly different from day 2 to day 14 (ns = not significant, univariate n-way ANOVA, Tukey's post-hoc test).

A

Start value element bounds	
CPE-P	Fixed day 2 EIS value
CPE-T	Fixed day 2 EIS value
R1	50 to 5000Ω
C	10^{-13} to 10^{-7}F
R2	Fixed day 2 EIS value
***fmincon* constraints**	
Start values	30 linear
Comb. start values	900
Max iterations	500
Step tolerance	10^{-12}

B

All days (n = 193)	
Total capped	60.6%
R1 & C capped	47.3%
R1 only capped	7.4%
C only capped	5.9%
**Day 9 to 14 (n = 68)**	
Total capped	21.2%
R1 & C capped	16.7%
R1 only capped	3.0%
C only capped	1.5%

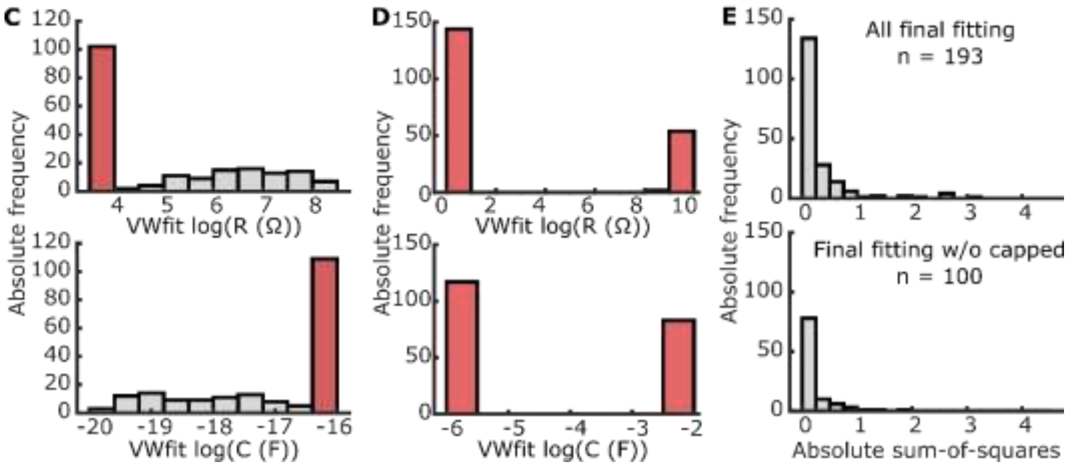

Figure S7. Constraints, capping, bimodal distributions and error of fit of reverse fitting of voltage waveforms to electrical circuit. **(A)** Start value element bounds and *fmincon* (MATLAB) constraints. **(B)** Percentage of capped values for all timepoints and for day 9 to 14. **(C)** Histogram of VW-fitted elements R_1 (top) and C (bottom) showing a clear bimodal distribution. The values in red are defined as capped, while the grey values are included in the analysis in Fig. 6D. **(D)** Histogram of VW-fitted elements R_1 (top) and C (bottom) with widened element bounds showing almost only capped values. **(E)** Histogram of absolute sum-of-squares for all VW-fittings (top) and for fittings without capped values (bottom).

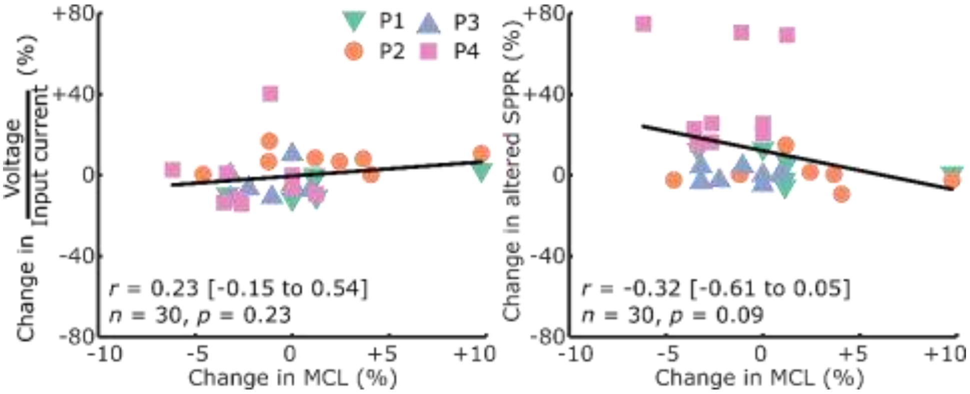

Figure S8. Contact 'impedances' and altered SPPR of 4 recently implanted CI patients and their correlation to mean comfortable loudness (MCL) levels. Change in contact 'impedances' (left) and altered SPPR (right) are calculated as the percentage difference at 5mo compared to the earlier 3mo timepoint for each electrode in each patient. No significant correlations at group level were found (Pearson's correlation coefficient).

	P1							P2							P3							P4						
	Contact 'impedance' (kΩ)			Altered SPPR (%)		MCL (CL)		Contact 'impedance' (kΩ)			Altered SPPR (%)		MCL (CL)		Contact 'impedance' (kΩ)			Altered SPPR (%)		MCL (CL)		Contact 'impedance' (kΩ)			Altered SPPR (%)		MCL (CL)	
Electrode number	Intra-op	3mo post-op	5mo post-op	3mo post-op	5mo post-op	3mo post-op	5mo post-op	Intra-op	3mo post-op	5mo post-op	3mo post-op	5mo post-op	3mo post-op	5mo post-op	Intra-op	3mo post-op	5mo post-op	3mo post-op	5mo post-op	3mo post-op	5mo post-op	Intra-op	3mo post-op	5mo post-op	3mo post-op	5mo post-op	3mo post-op	5mo post-op
1	5.4	12.3	12.3	31.2	24.3	160		3.3	12.2	12.2	31.2	30.5	174	166	2.3	6.7	7.2	57.1	57.2			2.5	7.0	7.0	56.7	68.5	188	188
2	6.5	11.5	11.0	29.0	24.1			4.1	13.3	12.2	33.3	30.3			2.2	6.4	6.3	59.3	56.4			2.6	6.8	6.6	52.5	64.3		
3	6.1	10.2	10.0	48.4	43.5		190	3.8	13.5	13.0	43.3	39.7			2.1	8.2	9.5	0.0	4.7	216		2.8	6.2	6.7	38.9	55.4		172
4	6.6	10.6	11.2	48.1	45.4	184		4.5	11.7	11.8	48.6	44.1	148	154	2.1	5.6	5.2	55.8	56.2	216	218	3.0	5.5	7.7	33.3	56.9	178	176
5	6.7	10.9	10.6	50.2	51.5		188	5.2	10.2	10.7	44.8	34.9			2.4	5.6	5.6	52.9	55.6			3.0	5.3	5.7	33.4	58.2		
6	9.8	12.5	12.8	45.9	43.2			4.2	11.3	12.1	46.4	41.2			2.8	4.7	5.4	33.2	50.8			3.3	7.0	6.1	53.3	63.4		
7	8.0	11.7	11.5	51.3	48.5	176	178	4.6	10.2	10.9	46.7	46.9	164	162	2.8	4.5	4.3	41.7	41.7	202	202	3.4	7.0	7.1	56.5	65.1	178	172
8	8.0	10.4	11.0	52.3	48.4			4.0	10.8	10.7	45.2	43.3			2.9	5.2	4.5	46.7	41.6			3.7	5.7	5.0	26.8	48.9		
9	8.7	12.8	14.1	51.3	47.6			4.3	11.5	11.1	41.6	45.0			2.8	5.0	4.6	38.5	39.3			3.5	5.1	4.6	28.5	50.3		
10	9.6	13.8	13.8	50.0	49.0	178	180	4.0	10.0	10.7	42.6	43.3	164	168	2.8	5.8	5.2	44.7	46.7	192	190	3.7	5.4	5.0	39.9	50.2	172	172
11	8.5	15.6	15.8	49.0	50.8			4.0	10.9	11.6	45.5	46.9			2.7	6.2	6.1	53.5	50.0			3.8	5.5	4.8	37.6	46.3	170	164
12	7.8	12.7	13.4	47.4	50.0			4.6	11.1	12.4	45.1	44.1			2.8	7.1	6.9	51.8	52.2			4.0	5.4	4.8	33.5	53.9		
13	7.4	11.8	12.1	51.4	51.8	164	180	5.0	11.1	12.0	46.9	47.1	164	170	2.8	7.3	7.4	56.5	54.6	188	182	4.8	5.3	4.8	33.2	56.2	162	164
14	7.9	13.0	12.3	43.3	48.7			5.1	11.1	13.5	50.0	43.2			2.9	7.9	8.5	56.8	59.3			5.1	5.1	5.3	28.6	52.4		154
15	8.3	13.8	13.6	41.1	47.5			5.2	11.5	13.5	40.6	39.3			3.2	6.9	6.9	48.2	52.1			4.5	5.9	5.7	29.1	44.8		
16	8.5	14.0	12.5	45.8	48.7	160	162	5.1	10.6	11.8	46.6	45.5	164	180	3.0	7.6	6.7	47.9	50.0	188	182	3.4	5.5	5.7	26.9	46.9	162	152
17	8.5	14.7	12.8	45.2	49.8			4.8	12.0	12.3	48.6	44.3			3.0	7.3	6.9	42.9	46.5			3.6	7.4	6.3	30.2	44.5		
18	9.7	15.2	14.2	47.6	48.9			4.9	11.8	14.8	51.3	45.3			3.0	7.1	7.6	43.4	46.1			3.8	8.5	7.6	37.5	43.3		
19	9.4	15.8	14.3	45.4	51.2	150	145	4.7	11.8	13.8	47.0	47.2	170	168	2.9	6.5	7.1	50.0	47.6	176	176	3.7	6.1	5.2	25.0	31.4	152	148
20	9.8	16.9	15.4	46.9	47.8			5.0	14.1	10.7	36.9	35.7			2.9	6.6	6.3	33.4	36.2			3.5	6.8	7.0	22.2	34.0		
21	10.2	16.3	15.6	47.9	51.0			7.2	10.2	8.7	33.5	34.8			2.7	6.5	6.2	44.5	36.9			4.0	7.2	7.0	26.1	39.0		
22	9.9	16.7	14.7	45.8	51.9	146	146	6.8	10.9	11.8	42.1	48.4	170	172	3.9	9.5	8.9	47.4	46.2	178	174	4.1	8.2	7.3	31.8	37.0	150	146

Table S1. Overview of patient data.
